# Zinc Complexes with Nitrogen Donor Ligands as Anticancer Agents

**DOI:** 10.3390/molecules25245814

**Published:** 2020-12-09

**Authors:** Marina Porchia, Maura Pellei, Fabio Del Bello, Carlo Santini

**Affiliations:** 1 ICMATE-C.N.R., Corso Stati Uniti 4, 35127 Padova, Italy; 2 Chemistry Division, School of Science and Technology, University of Camerino, via S. Agostino 1, 62032 Camerino, Italy; carlo.santini@unicam.it; 3 Medicinal Chemistry Unit, School of Pharmacy, University of Camerino, Via S. Agostino 1, 62032 Camerino, Italy; fabio.delbello@unicam.it

**Keywords:** zinc(II), zinc(II) complexes, N-donor ligands, medicinal chemistry, antitumor agents

## Abstract

The search for anticancer metal-based drugs alternative to platinum derivatives could not exclude zinc derivatives due to the importance of this metal for the correct functioning of the human body. Zinc, the second most abundant trace element in the human body, is one of the most important micro-elements essential for human physiology. Its ubiquity in thousands of proteins and enzymes is related to its chemical features, in particular its lack of redox activity and its ability to support different coordination geometries and to promote fast ligands exchange. Analogously to other trace elements, the impairment of its homeostasis can lead to various diseases and in some cases can be also related to cancer development. However, in addition to its physiological role, zinc can have beneficial therapeutic and preventive effects on infectious diseases and, compared to other metal-based drugs, Zn(II) complexes generally exert lower toxicity and offer few side effects. Zinc derivatives have been proposed as antitumor agents and, among the great number of zinc coordination complexes which have been described so far, this review focuses on the design, synthesis and biological studies of zinc complexes comprising N-donor ligands and that have been reported within the last five years.

## 1. Introduction

Zinc is among the few transition metals, namely Mn, Fe, Co, Cu, Zn and Mo, which, together with the first and second series metals Na, K, Mg and Ca, are essential for human physiology. In the human body, zinc, after iron, is the second most abundant trace element. About 3 g of zinc, mostly localized in testicles, muscles, liver, and brain, are present in an average adult provided by a daily intake of 8–11 mg [1]. At the physiological concentration, zinc is crucial for increasing cell survival and protecting tissues against damages. Zinc concentration (about 0.6 mM) is regulated by a specific homeostasis and, similarly to the other micro-elements, either a deficiency or an overload can lead to toxic effects to the organism [2,3,4,5]. Zinc deficiency can be related to inadequate zinc intake due to nutritional or absorption problems, ageing (several data showed that 35–45% of adults over 60 have a Zn intake below the required estimated average), Zn losses from the body or deregulation of zinc homeostasis. Zinc deficiency can depress immune function as Zn plays a crucial role in the immune system through cellular proliferation and RNA and DNA synthesis and is necessary for T-lymphocyte development. It also can determine other effects including growth retardation, impotence, and hypogonadism. Many symptoms due to Zn deficiency are not specific and can be related to other health conditions, so that the diagnosis is not always straightforward. Zinc excess, however, is less frequent and most often occurs via excess supplementation. Most toxic effects due to a chronic high Zn intake (e.g., myeloneuropathy) are mainly related to the inhibition of copper absorption, and hence are secondary to a zinc-induced copper deficiency [6].

The vital importance of zinc can be easily understood considering that this metal is present in more than 3000 human proteins including nucleic acid binding proteins; is involved in the catalytic activity of thousands of enzymes; plays a role in DNA synthesis, protein synthesis and immune functions [7,8]. The binding of Zn^2+^ with catalytic and/or structural sites of a large number of proteins is a key-factor in determining their conformations [2]. All in all, zinc is essential for virtually all cellular functions and also for the growth and development of all forms of life, not only human [9]. The majority of Zn in the human body (95%) is intracellular and the lack of specialized zinc storage systems makes a suitable daily intake necessary for maintaining a steady concentration in the organism. In biological systems the concentration of free Zn^2+^ ions is extremely low (pM-nM), i.e., it is not a relevant pool for trafficking, transport and cellular actions of zinc, so that these processes occur by a direct exchange of the metal from donor to acceptor Zn ligands [10]. Specific Zn transporters (ZIP and ZnT proteins) regulate Zn homeostasis and control its efflux via plasma membranes when the concentration of intracellular Zn is too high or when it must be transferred to other organs. But, whereas zinc coordination and its role in proteins and enzymes has been clarified and extensively reviewed, further studies are still necessary to completely explain the mechanisms of the exchange processes between intra and extracellular space [11].

The importance of zinc in biological systems is definitively related to its unique chemical features: Zn^2+^ is redox inactive, is a strong Lewis acid, has a d^10^ configuration, is diamagnetic, can support a variable coordination geometry and is prone to a fast exchange of ligands. Its electron affinity resembles that of copper or nickel, but the lack of redox activity of divalent zinc ion, differently from copper or iron, eliminates any chances of free radical reactions and makes it crucial for the body’s antioxidant protection system.

The Zn^2+^ d^10^ configuration, and the consequent absence of d-d transition, could be seen as a limit for the spectroscopic characterization of Zn derivatives, together with their diamagnetism and white colour, but on the other hand the absence of ligand field stabilization can guarantee highly flexible coordination geometry determinated only by the charge and steric hindrance of the ligands [12,13]. In biological systems zinc can be tetra-, penta-, or hexacoordinated to N, O or S donor atoms comprised in histidine, glutamate/aspartate, and cysteine residues, or to water molecules with a tetrahedral, pyramidal, or octahedral coordination geometry. In proteins, the most frequent geometry is tetrahedral, with few examples of distorted trigonal bipyramidal. In proteins, also multiple zinc clusters, comprising from two to four metal ions, can be found in the metal intrasphere binding geometry.

In addition to its physiological role, zinc can have beneficial therapeutic and preventive effects on infectious diseases and, compared to other metal-based drugs, Zn(II) complexes generally exert lower toxicity and have fewer side effects. An example of a worldwide commercial Zn-derivative is pyrithione zinc, first described in 1930 and used as topical antimicrobial to treat fungal or bacterial infections of skin and hair. In the years different classes of zinc coordination complexes have shown a good potential in different applications, among which as radioprotective agents [14], tumor photosensitizers [15], antidiabetic [16,17,18], anticonvulsant [19], anti-inflammatory [20], antimicrobial [21,22,23,24,25,26], antioxidant [27,28], antiproliferative/antitumor [29,30,31], anti-Alzheimer’s disease [32] and in several neglected diseases [33].

On the other hand, deregulation of zinc homeostasis can determine cell apoptosis and hence trigger cancer progress [34]. The relationship between zinc deficiency and cancer has been recognized in human, animal, and cell culture studies [35,36] and zinc-containing metalloenzymes have been identified as alternative targets for metal-based anticancer agents [37]. Zinc deficiency causes oxidative DNA damage [38,39], and chromosome breaks have been reported in zinc-deficient diet-fed animals [40]. In addition, zinc is useful in reducing cardio and hepatotoxicity caused by some anticancer drugs [41].

The relationship between Zn deficiency and prostate cancer has been deeply analysed [42], as well as the effect that Zn imbalance can have on the genesis and development of different forms of leukemia [43].

Examples of the detrimental effects of both excess or depletion of Zn in tumoral pathologies have been faced with opposite approaches: on one hand a chelation therapy approach based on depletion of excess cellular Zn by the use of suitable chelating ligands [44,45], on the other hand the use of ionophore systems such as clioquinol [46].

Another approach consists in using zinc complexes as metal-based antitumor drugs. This approach is very promising due to the fact that (1) having a specific homeostasis zinc metal ion could be better managed by human physiology and cause less side effects in comparison to non-essential metal-based compounds [47,48]; (2) zinc is significantly non-toxic even at higher doses than other metals (Fe, Cu, Hg, etc.), with obvious advantages for bio-compatibility [47,48,49,50,51]; (3) Zn(II) complexes probably have targets and mechanisms of action different from the classical platinum-based drugs [52,53,54,55]; (4) zinc is one of the most studied metals in the coordination of photosensitive systems metals for Photo Dynamic Therapy (PDT) [56,57,58], and (5) due to their ability to assist Lewis activation, nucleophile formation and rapid ligand exchange, zinc compounds can be employed as catalysts of hydrolytic reactions, such as hydrolysis and DNA cleavage, thus making anti-tumor activity possible [59,60]. Recent studies have confirmed the above assumptions showing that Zn(II) derivatives could be potential anticancer agents with low toxicity in vivo, low side effects and probably different cellular targets and modes of action when compared with classical metal-based drugs [53,55,61,62,63,64,65,66].

A large variety of zinc complexes containing ligands of different hapticity with mainly O, N and S as donor atoms, exhibiting different coordination numbers and geometries, often giving rise to dimeric or polymeric species have been reported. As, to the best of our knowledge, no recent review article on zinc complexes as anticancer agents is available and taking into account that in biological systems nitrogen is the most utilized atom for Zn coordination, we started to survey zinc complexes which comprise N-donor ligands. The aim of the present review article is to describe the development in the synthesis, design, and biological studies of zinc complexes of N-donor ligands as anticancer agents covering the period 2015–2019. All other classes of zinc complexes will be reported in a following article. The compounds (102 entries) are grouped based on the ligand donor atom set, by increasing ligand complexity. We tried to identify possible structure-activity relationships (SARs) for each class of ligands described and, by a critical analysis of the reported data, to indicate the new directions of the research for scientists working in this field. The analysis of mechanistic details exceeds the scopes of this review.

## 2. Nitrogen Ligands in Zn Complexes

As reported in the Introduction, Zn(II) has a very versatile chemistry. It can adopt a range of coordination numbers giving rise to different geometry, even though especially in solution octahedral stereochemistries dominate. Zinc can coordinate various donor atoms, especially the first-row donor atoms oxygen or nitrogen rather than second-row sulphur or phosphorus, according to its hard acid nature. Accordingly, N-donor ligands are almost the most representative category. Homoleptic and mixed-ligand complexes have been reported and, due to the variety of accessible arrangements, a great assortment of frameworks (from mono- to hexadentate chelates) have been employed.

Our classification is based on the ligand nature. Planar aromatic quinoline, 2,2′-bipyridine and 1,10-phenanthroline ligands have often been the ligands of choice for medicinal chemists, due to their DNA intercalation properties and often to their intrinsic toxicity, which could enhance the metal effect. On the other hand it’s known that several diimines have low specificity for tumor cell lines and can be genotoxic [67]. Here we report on 24 Zn complexes with these ligands (cap. 3 and 4). Terpyridine metal complexes are able to intercalate into DNA showing inhibitory effects on tumor cells and possess photoluminescence properties [68,69,70,71]. Here we report on 23 Zn complexes with terpyridine and pyridine-based systems (cap. 5). Imidazolyl derivatives are among the most utilized N-donor ligands due to their excellent coordination ability [72,73], different hapticity and possibility to be derivatized or conjugated to active moieties. Within this class, benzoimidazolyl derivatives are the most representative (22 out of 36 Zn complexes, cap. 6), mainly thanks to the accessibility of phenyl ring substitution, which in turn allows SAR determination for different families of Zn complexes. Schiff bases are generally one of the most representative class of ligands, mainly due to their easy way of synthesis. N-donor Schiff bases have been surveyed on the basis of their different hapticity (9 Zn complexes, cap. 7), whereas some examples of *N*,*O*-coordination are reported in the miscellanea (10 Zn complexes, cap. 8).

Another application of Zn derivatives in medicinal chemistry is Photo Dynamic Therapy and, among many metals utilized in the coordination of photosensitive systems, such as phthalocyanines, zinc is one of the most studied. Zinc complexes with photo-activable N-donor ligands, such as porphyrins and phthalocyanines, used in PDT, have been extensively reviewed in the last years [56,57] and are not treated in this paper. Zinc-phthalocyanine complexes generally show low toxicity, high chemical and photochemical stability [56]. Anyway, low dark cytotoxicity is generally a prerequisite for photosensitizers in biological applications, even though chemotoxicity is sometimes associated to some Zn derivatives. The phototoxicity of the reported complexes is generally very high (IC_50_ values in micro-nanomolar range) and cannot be compared to the toxicity showed by the other families of zinc complexes surveyed in this paper, as the mechanism of action is not relied upon a biological involvement of the metal.

## 3. Quinoline and Diimine Systems

**Figure 1:**
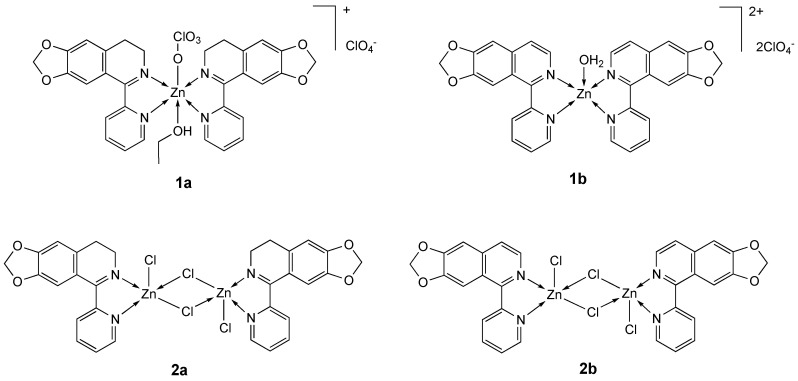
Structure of the zinc complexes **1a**, **1b** and **2a**, **2b**.

The planar aromatic ligands 4,5-methylenedioxy-1-pyridinedihydroisoquinoline **L_1a_** and 5-pyridin-2-yl-[1,3]dioxolo [4,5-g]isoquinoline) **L_1b_** have been used for the synthesis of monomeric **1a** and **1b** [74] and binuclear **2a** and **2b** complexes [75] (Figure 1). The new complexes have been structurally characterized and tested against a panel of tumor as well as normal cell lines.

All Zn derivatives showed a remarkable anticancer activity and selectivity to tumor cells; in particular binuclear **2a** and **2b** complexes are more active than mononuclear complexes **1a** and **1b**; penta-coordinated complexes are more active than hexacoordinate ones and species comprising the **L_1b_** ligand are more active than **L_1a_** complexes (Table 1). Studies on complex **1b** showed a noticeable cellular uptake and DNA accumulation, and DNA interaction via an intercalating mode. In vitro test with MGC-803 cell line with the most potent compound **2b** evidenced that it has a good DNA accumulation and cellular uptake and induces the intrinsic pathway-dependent apoptosis by triggering DNA damage due to reactive oxygen species (ROS) overproduction [74,75].

## 4. 2,2′-Bipyridine and 1,10-Phenanthroline Systems

Considering DNA the main target of Zn metal-based drugs, a widespread use of planar intercalating systems, like diimine, as ligands has been done. In particular, 2,2′-bipyridine and 1,10-phenanthroline derivatives have been extensively utilized often together with other nitrogen or oxygen donor ligands.

1,10-Phenanthroline (phen) is a versatile nitrogen-chelating bidentate ligand [76] based on rigid electron-deficient heteroaromatic rings, which displays strong cooperativity in cation binding to form stable transition metal complexes in solution. Phen, due to its strong hydrophobic interaction and large plate area, heads-up intercalation or groove binding with DNA or RNA of the related metal complexes [77,78,79,80,81,82].

In contrast to phen, 2,2′-bipyridine (bpy) has been extensively employed as chelating ligand due to its strong redox stability and the possibility of functionalization [83,84].

Zn(II) [85], Cu(II) [86,87,88,89,90,91,92], V(IV) [93,94], and Ru(II) [95,96] complexes with polypyridyl ligands have been intensively investigated as DNA intercalators, showing a diverse spectrum of DNA binding/cleavage ability and cytotoxicity against cancer cell lines [97].

**Figure 2:**
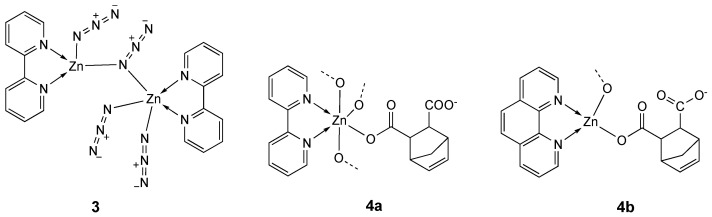
Structure of the zinc 2,2′-bipyridine complexes **3** and **4a**, **4b**.

The water-soluble dimer **3** (Figure 2) of 2,2′-bipyridine and azide ligands comprises the Zn atoms coordinated by two nitrogen atoms from a 2,2′-bipyridine ligand, two nitrogen atoms from two azido bridges and one terminal azide nitrogen [98] in a distorted square pyramidal geometry [99]. Complex **3** cleaves DNA via hydrolytic pathway (T4DNA ligase assay), but its antiproliferative activity tested in vitro on MCF-7 breast cancer cell line was low (IC_50_ = 100 µM) (Table 2).

Other two complexes (**4a** and **4b**, Figure 2) have been synthesized and characterized by Enjun Gao et al. [100]. The coordination environment of Zn atom in **4a**, an example of a 1D spiral-like network, is a distorted ZnO_4_N_2_ octahedral geometry, whereas in **4b**, an infinite double-stranded helix, Zn is in a distorted ZnO_2_N_2_ tetrahedral geometry.

The cytotoxic effects of complexes **4a** and **4b** were studied on two tumor cell lines (HeLa and KB) and one normal cell line (LO-2). In particular, **4b** showed IC_50_ values comparable to the reference cisplatin (IC_50_ = 12.4 and 15.2 µM; cisplatin, IC_50_ = 11.9 and 13.8 µM; for HeLa and KB cells, respectively) and both Zn derivatives were less toxic against the normal cell line than cisplatin (Table 2). The phen-derivative **4b** showed also a better cell uptake efficiency and binding with Fish Sperm DNA [100].

**Figure 3:**
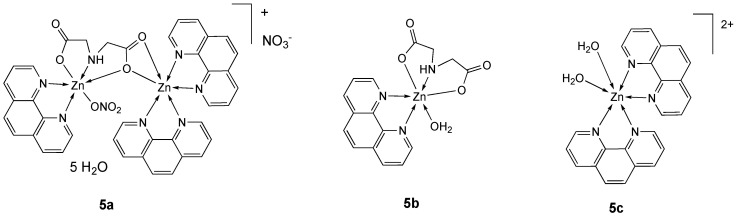
Structure of the zinc complexes **5a**–**c**.

The use of an iminodiacetate ligand (ida) [104,105] and phen led to the formation of a water soluble asymmetric binuclear zinc(II) complex **5a**, which in aqueous solution dissociates into the two monomeric species **5b** and **5c** (Figure 3), as assessed by NMR, ESI-MS, and solution UV-vis spectra [101]. The in vitro cytotoxicity of the zinc complexes **5a**–**c**, a 1/1 mixture of **5b** and **5c**, free ligands and zinc salts (ZnCl_2_ and ZnSO_4_) were investigated in human hepatoma HepG2 and SMMC-7721 cell lines.

As shown in Table 2, the cytotoxic activities of the tested compounds are in the following order: **5a** ≈ mixture 1:1 (**5b** + **5c**) > **5c** > **5b** > ida, phen > ZnCl_2_, ZnSO_4_. From these data, it’s evident that the binuclear **5a** mainly acts as a cooperative inhibitor with complexes **5b** and **5c** toward tumor growth in solution; its activity is related to mainly arrest the cell cycle at G0/G1 phase.

Complex **5a** is one of the few examples of zinc derivatives tested in in vivo studies. In particular, the acute toxicity of the oral administration of **5a** in ICR mice was studied, obtaining an LD_50_ value of 736 mg kg^−1^ (with the 95% confidence limit of 635–842 mg kg^−1^), indicative of its low toxicity.

A series of first-row transition-metal compounds (M = Co(II), Ni(II), Cu(II) and Zn(II)) comprising a diimine (bpy or phen) and the ligand norharmane (Hnor) [106,107,108,109] have been reported [102].

**Figure 4:**
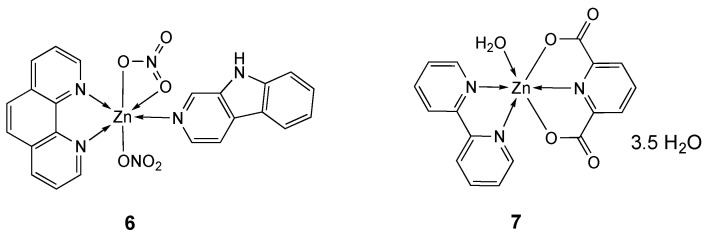
Structure of the zinc complexes **6** and **7**.

The antitumor activity of complex **6** (Figure 4) was determined with and without co-incubation with CuCl_2_, a competitor for transport via hCTR1 (CuCl_2_ 20 µM, the highest non-toxic dose), against A2780 tumor cell line. Whereas co-incubation of CuCl_2_ drastically increased the cytotoxicity of Co(II) derivatives, probably due to the replacement in solution of the metal with copper leading to the most stable Cu complexes, which are endowed with enhanced antiproliferative activity, in the case of **6**, already per se very cytotoxic (IC_50_ = 1.26 ± 0.07 µM), no effects could be recorded (**6** + CuCl_2_, IC_50_ = 1.23 ± 0.01µM) (Table 2) [102].

The octahedral bpy derivative **7** (Figure 4), containing 2,6-pyridine dicarboxylate (pdc) [110,111], inhibited cell viability by inducing apoptotic cell death in T-cell lymphoma cancer cell line (IC_50_ = 17.12 µM) with negligible cytotoxicity in PBMC normal cells (IC_50_ = 63.23 µM) (Table 2). The molecular docking and SAR studies showed that **7** was effective in inhibiting highly expressed cancer target proteins [103].

**Figure 5:**
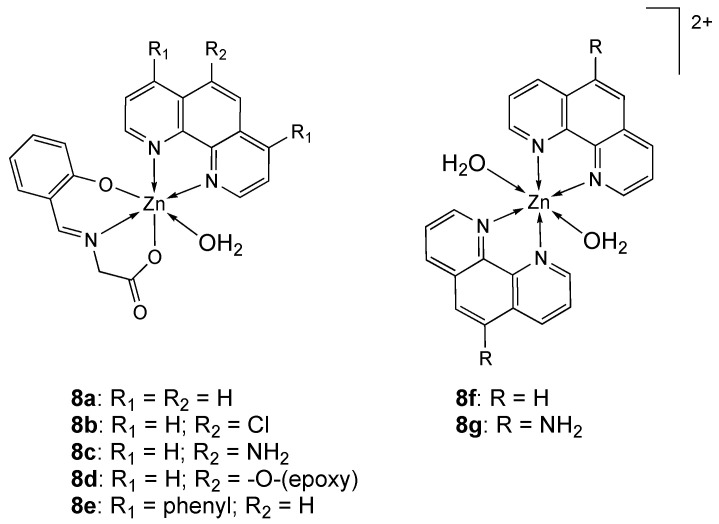
Structure of the zinc complexes **8a**–**g**.

Mixed-ligand zinc complexes with *N*-salicylideneglycinate (Sal-Gly) [86,93,112] and 1,10-phenanthroline (phen) (**8a**, Figure 5) or phenanthroline derivatives 5-chloro-1,10-phenanthroline (Clphen), 5-amine-1,10-phenanthroline (amphen), 4,7-diphenyl-1,10-phenanthroline (Bphen), 5,6-epoxy-5,6-dihydro-1,10-phenanthroline (epoxyphen) (**8b**–**e**, Figure 5), as well as homoleptic zinc complexes **8f**,**g** (Figure 5) have been screened for finding a relationship between their biological activity and the nature of phen-derivative [113].

Their cytotoxicity was evaluated towards a panel of human cancer cell lines (A2780, MCF-7 and HeLa), as well as non-tumor V79 fibroblasts. As shown in Table 3, all complexes and free ligands displayed high cytotoxicity, better than or comparable to the reference cisplatin, with IC_50_ values in the low micromolar range. They showed the following cytotoxicity order: A2780 > MCF-7 > HeLa. Overall, all compounds displayed selectivity for the A2780 cells with respect to the non-tumor cells, with a selectivity indexes (SI = IC_50_(V79)/IC_50_(A2780) > 2) higher than cisplatin (SI = 1).

All complexes induced caspase-dependent apoptosis in A2780 cells, except **8e**, one of the most cytotoxic within the series. The [Zn(Sal-Gly)_2_(H_2_O)_2_] precursor showed no cytotoxicity, highlighting the active role of the polypyridyls to the biological activity of the complexes. The free ligands displayed cytotoxic activity similar or even higher than the complexes, suggesting that the active species are the free phen-derivatives even though, as shown by different experiments, their uptake may possibly be promoted or hampered by their binding to Zn(II) and/or albumin. The IC_50_ values obtained for **8f** and **8g** in all cancer cells were about half the values obtained for **8a** and **8c** complexes. Considering that each zinc 1,10-phenanthroline complex contains two moles of 1,10-phenanthrolines, this suggests that the active species is the phen ligand [85,118,119]. In addition, no cytotoxic activity was observed for the ZnCl_2_ salt (IC_50_ > 100 μM) in the ovarian cells during 48 h incubation in the same concentration range of the complexes (0.01 μM−100 μM). These results are in accordance with the reported statement that Zn(II), due to its hydrophilicity, cannot cross the plasma membrane or other membranes of intracellular components to induce a biological effect [120]. Zn-complexes undergo speciation in the cell incubation medium; anyway complexation with zinc may be relevant, since Zn inside the cell can also interfere with the metabolism and the mitochondrial electron transport to generate ROS [113].

**Figure 6:**
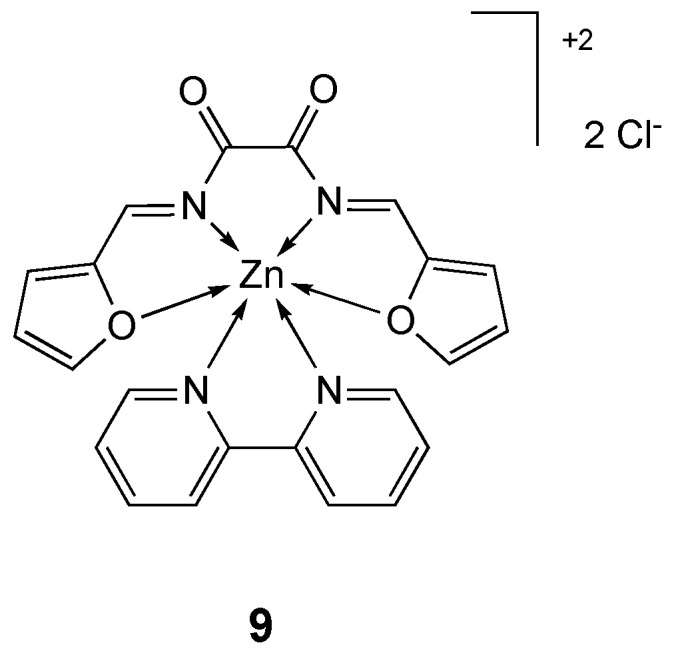
Structure of the zinc complex **9**.

Mixed ligand complexes of the Schiff base ligand **L_9_** and 2,2′-bipyridine and several transition metals (Cr(III), Fe(III), Cu(II), Cd(II), Mn(II), Co(II), Ni(II) and Zn(II)), were synthesized [114] and their cytotoxicity was tested against breast cancer (MCF-7) and colon cancer (HCT-116) cell lines (Table 3). The free ligand **L_9_** resulted inactive (inhibition ratio < 70%), while the complexes showed much higher activity against MCF-7 cell line with the following order: Fe(III) > Cd(II) > Cr(III) > Ni(II) > **9** > 2,2′-bipyridine > Mn(II) (Figure 6). On the other hand, free ligand and mixed ligand complexes show moderate to high activity against HCT-116 cell line with the following order: Co(II) > Cr(III) > Ni(II) > 2,2′-bipyridine > Mn(II) > Fe(III) > **L_9_** > **9**.

**Figure 7:**
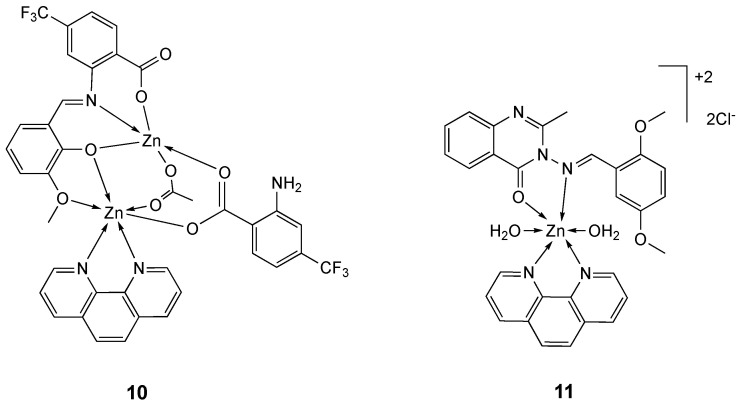
Structure of the zinc complexes **10** and **11**.

The zinc complex **10** (Figure 7) has been synthesized and characterized by spectroscopic techniques and X-ray crystallography. Its cytotoxic effect on A549 and HeLa cancer cells was screened. Results showed that **10** exhibits high antitumor activity against both A549 and HeLa cell lines, with IC_50_ values of 1.369 µM and 2.129 µM, respectively (Table 3) [115]. Analogously to what observed for complexes **8a**–**g**, the high toxicity might be due to the phen ligand. This result may also be attributed to the high compound lipophilicity, which enhances its ability to cross the cell membrane. It is speculated that the fluorine-containing zinc complex has a better prospect in the development of antitumor drugs [121,122].

The bidentate quinolin-4(3*H*)-one based Schiff base ligand **L_11_** and its 1,10-phenanthroline zinc complex **11** (Figure 7) have been screened for their in vitro cytotoxic activity against the human breast cancer MCF-7 cell line [116]. Even if the antiproliferative activity of metal complexes is often due to the synergistic effect produced by the metal core and the ligands, the free ligand **L_11_** resulted inactive against MCF-7 cells. On the basis of these findings, the noteworthy anticancer activity of **11** may be due to phen moiety or to the metal center zinc(II). In fact, complex **11** exhibited interesting anticancer activity against MCF-7 cells (GI_50_ = 0.016 μM) even at lower GI_50_ value than the reference drug doxorubicin (GI_50_ = 0.018 μM) (Table 3). In addition, the cells treated with **11** displayed a bead like shape, indicating cellular shrinkage, vacuolated cytoplasm, small nuclei and membrionic blebbing that are typical features of apoptosis [116].

**Figure 8:**
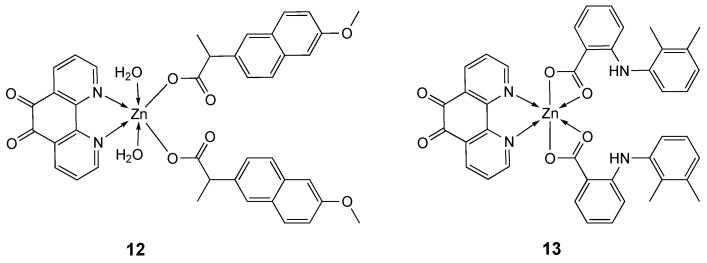
Structure of the zinc-NSAID complexes **12** and **13** with naproxen and mefenamic acid, respectively.

Metal-NSAID (NSAID = non-steroidal anti-inflammatory drug) complexes can show a synergistic anticancer effect [123,124] and, in recent times, their non-platinum metal complexes have exhibited anti-proliferative as well as anti-inflammatory activities [125,126,127,128,129,130]. Jolly Deb et al. [117] reported the synthesis of two zinc(II)-NSAID complexes of 1,10-phenanthroline-5,6-dione (phendione) [82,126,131,132], with the NASAID species naproxen (HNPR) and mefenamic acid (HMFN) (**12** and **13**, respectively, Figure 8).

The bidentate chelating ligand phendione has been reported to exhibit antiproliferative activity and to interact with DNA by its aromatic ring. The cytotoxic activities of the compounds were determined on cisplatin resistant MDA-MB-231 cancer cell line and for comparison on non-cancerous mouse macrophage cell line RAW 264.7. The complexes **12** and **13** exhibited cytotoxic activity on MDA-MB-231 cells with IC_50_ values after 72 h of 0.5 μM and 0.4 μM, respectively (Table 3), and were less toxic in the RAW 264.7 cell line (with IC_50_ values after 72 h of 2 μM and 1.7 μM, respectively). Whereas free NaNPR (sodium naproxen), HMFN, and [Zn (ClO_4_)_2_]·6H_2_O did not have any significant antiproliferative properties, co-treatment with free phendione and naproxen or mefenamic acid (1:2 ratio) determined in both case an IC_50_ value of 0.4 μM, confirming that phendione acts independently and that the anti-proliferative properties of the Zn ternary complexes are due to the phendione unit. Complexes **12** and **13** explicated a dual activity, as they also inhibited the cyclooxygenase pathway exhibiting anti-inflammatory activity [117].

## 5. Terpyridine and Pyridine-Based Systems

Terpyridine metal complexes are gaining wide attention both for their ability to intercalate into DNA, and hence for inhibitory effects on tumor cells, and for their photoluminescence properties, that make them potential fluorescent materials or bioprobes [68,69,70,71,133,134,135,136,137,138,139,140].

**Figure 9:**
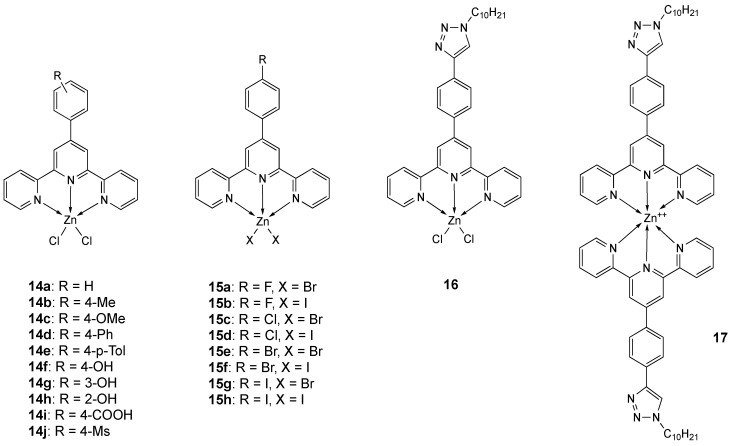
Structure of the zinc complexes **14**–**17**.

Extremely active penta-coordinated zinc complexes **14a**–**j** [141] and **15a**–**h** [136] (Figure 9) have been obtained by using terpyridine derivatives as ligands.

Different substituents on the terpyridine phenyl ring allowed to fine tune the lipophilicity and the steric hindrance of the final complexes, in order to find a possible SAR. All **14a**–**j** compounds display photoluminescent properties, the intensity of fluorescence emission peaks decreasing by CT-DNA interaction. Intercalation into the base pairs of DNA is confirmed by molecular docking studies. The IC_50_ values are in the sub-micromolar range, depending on the nature of the terpyridine ligand. In the case of **15a**–**h** derivatives (Figure 9), the hydrophilicity/lipophilicity ratio of the final complexes shows a range of distribution coefficients (log D7.4) between 0.31 (complex **15h**) and 0.87 (complex **15a**). The lipophilicity of the compounds depends on the nature of the halogen and is greater with bromine ions than with iodine ones, halogen substituents being equal. The remarkable antiproliferative activity of all complexes in MCF-7 and Bel-7042 cell lines can be related to the electronegativity of the substituted halogens or of the halogen anions and the same trend is observed in their binding affinity to ctDNA. In particular, the highest antitumor activity is shown by the fluorine substituted compounds, while the lowest is shown by the iodine substituted ones, following the sequence –F, –Cl, –Br, and –I substituents [136].

In Table 4, IC_50_ values obtained with compounds **14a**–**j** and **15a**–**h** are reported together to those of the penta-coordinated complex **16** (Figure 9), the hexacoordinated complex **17** (Figure 9) and the free ligand **L_16_** [142]. In the ligand **L_16_** the terpyridine scaffold was modified by inserting a long chain to increase its lipophilicity, but the activity of the free ligand was similar or even higher than that of Zn complexes, according with its DNA intercalation and cleavage ability [142].

**Figure 10:**
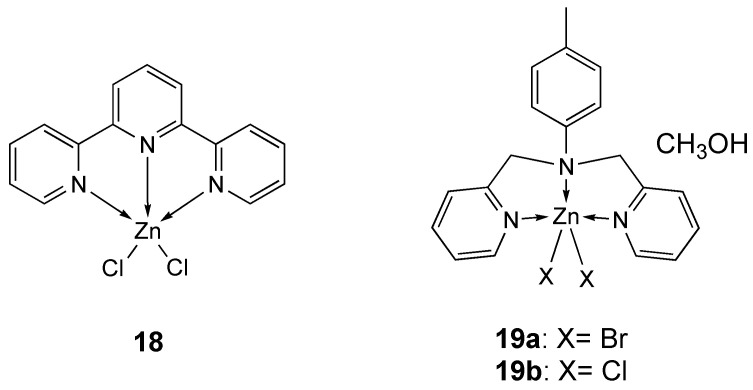
Structure of the zinc complexes **18** and **19a**, **19b**.

A penta-coordinated terpyridine (**18**, Figure 10) has been reported and its cytotoxic activity was evaluated [143], resulting significantly cytotoxic on MDA-MB-231 after 72 h (IC_50_ = 23 µM), and on HCT-116 after 24 h (IC_50_ = 10 µM) (Table 4). Its activity can be correlated both with its square-pyramidal structure, less susceptible to changes in coordination geometry in solution with respect to a tetrahedral structure (e.g., the analogous ethylenediamine complex), and to the planar terpyridine ligand which can act as an intercalator [143].

Following previous studies on zinc complexes with the tridentate polypyridyl ligand 4-methyl-*N*,*N*-bis(pyridin-2-ylmethyl)aniline (**L_19_**) [66], Yong-Po Zhang et al. reported the synthesis and structural characterization of [ZnL_19_X_2_]_2_·CH_3_OH (X = Br for 1**9a**, Cl for **19b**) [144] (Figure 10). Zinc atom is penta-coordinated in a square pyramidal environment.

Their antitumor activity has been investigated towards three human cancer cell lines (HeLa, MCF-7 and RL952). As reported in Table 4, both compounds showed good antitumor activity and the best results were obtained with **19a** (Figure 10) on MCF-7 cell line (IC_50_ = 12.58 µM) and with **19b** (Figure 10) on RL952 cells (IC_50_ = 11.71 µM). Moreover, both compounds in human normal liver cells LO2 showed lower cytotoxicity than cisplatin (**19a**: IC_50_ = 46.4 ± 1.4 µM; **19b**: IC_50_ = 40.2 ± 1.7 µM; cisplatin: IC_50_ = 9.6 ± 0.4 µM). The apoptosis-inducing activity of **19a** was assessed in cell morphology by nuclear staining with Hoechst 33,342, Annexin V binding studies and cell cycle analyses [144].

## 6. Imidazoles and Analogous Imidazole-Based Systems

**Figure 11:**
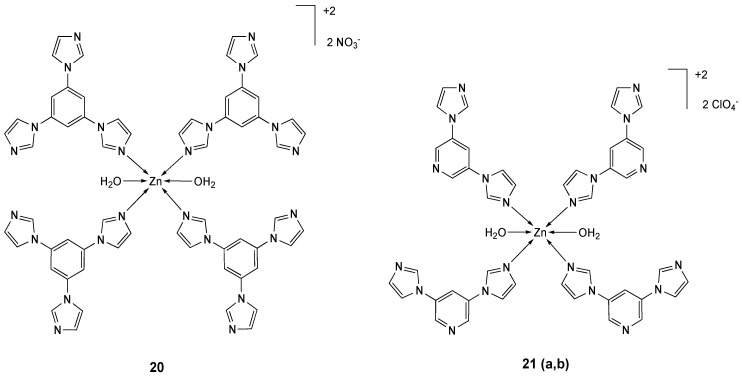
Structure of the zinc complexes **20** and **21**.

The octahedral compound **20** (Figure 11) derived from 1,3,5-tris(1-imidazolyl)benzene (H_2_tib [145,146]) [147] and two isomeric zinc complexes **21a** and **21b** (Figure 11) comprising 3,5-bis(1-imidazoly)pyridine (bip) [148] have been reported and structurally characterized. All compounds showed a remarkable activity against HeLa cell line, comparable to cisplatin (Table 5).

All compounds interact with DNA with different binding affinities and exhibit an efficient DNA cleavage (pBR322 plasmid). The better activity of isomer **21b**, compared to isomer **21a**, is explained by its parallel planar structure, which can be inserted into a DNA base pair as shown by molecular docking simulation.

**Figure 12:**
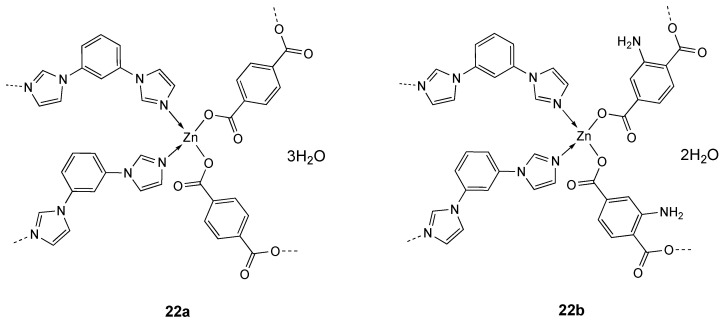
Structure of the zinc complexes **22a**, **22b**.

Other examples of Zn complex containing a monodentate bis-imidazolyl derivative are the Zn(II) coordination polymers **22a** and **22b** (Figure 12), containing the 1,4-dicarboxybenzene (H_2_bdc) and 1,3-bis(imidazol-1-yl)benzene (bib) or 2-amino-1,4-dicarboxybenzene (NH_2_-H_2_bdc), whose IC_50_ values calculated against SMMC-7721 liver cancer cells were 3.98 ± 0.11 and 9.78 ± 0.23 µg/mL, respectively (Table 6). Further studies on compound **22a** showed that its anticancer activity was due to the induction of ROS mediated cell apoptosis [149].

**Figure 13:**
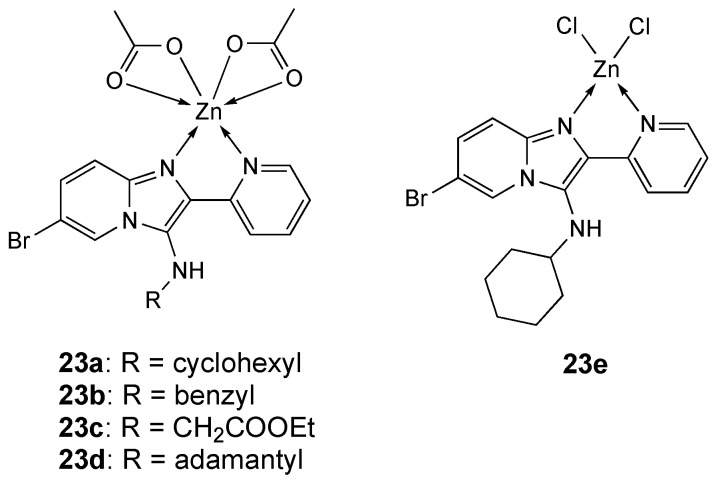
Structure of the zinc complexes **23a**–**e**.

Pyridine substituted imidazo[1,2-a]pyridines [150,151,152,153,154] were utilized for the preparation of a series of Cu and Zn complexes (**23a**–**e**, Figure 13).

Among them, zinc complexes **23c** and **23d**, tested against five cancer cell lines (MCF-7, MDA-MB-231, K562, HL-60 and HT29), exhibited poor activity (Table 6) compared to Cu derivatives (IC_50_ in the low micromolar range) [155].

**Figure 14:**
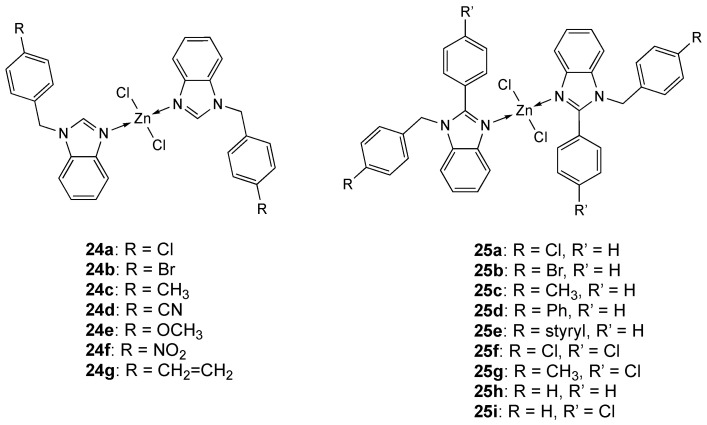
Structure of the zinc complexes **24a**–**g** and **25a**–**i**.

Many authors reported the use of substituted benzimidazole ligands [156,157,158] to obtain Zn complexes. In particular seven Zn derivatives **24a**–**g** (Figure 14) was reported by Elif Apohan et al. [159] and nine, **25a**–**i** (Figure 14), by Ülkü Yılmaz et al. [160].

In both studies, Zn ions have the same coordination sphere, as the metal is tetracoordinated to two nitrogen atoms of two different benzimidazole ligands and to two chloride ions. In the first series, the antiproliferative activity has been tested against A-549 tumor cell line and normal BEAS-2B cell line. At 72 h, zinc complexes showed a toxic effect against A-549 cells analogous to cisplatin, but they were less toxic than cisplatin on BEAS-2B (Table 7). Complexes **24a** and **24c**, containing 4-chlorobenzyl and 4-methylbenzyl substituents, were the most active derivatives (at 72h, IC_50_ = 1.97 and 1.9 µg/mL, respectively; cisplatin IC_50_ = 2.56 µg/mL). The second class of complexes **25a**–**i** was tested against A2780 and Du-145 tumor cell lines. At concentration of 0.1 μM compounds **25a**, **25b** and **25e,** containing 4-chlorobenzyl, 4-bromobenzyl or 4-styrylbenzyl substituent, respectively, showed anticancer activity against the A2780 cell line higher than the reference drug docetaxel (Table 7).

**Figure 15:**
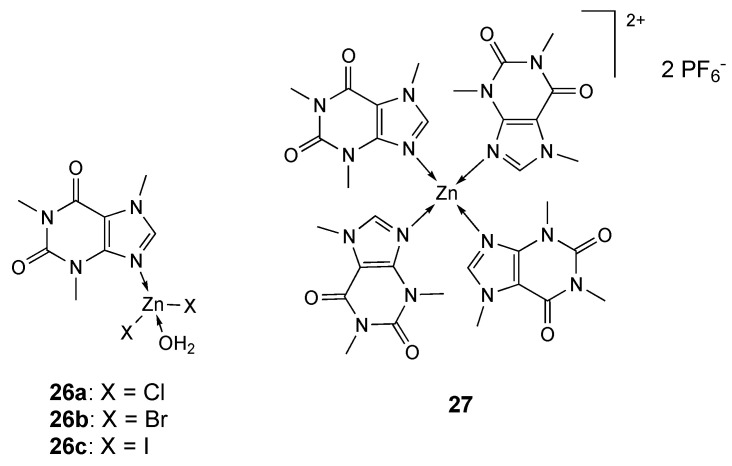
Structure of the zinc complexes **26a**–**c** and **27**.

A series of Zn-caffeine complexes (**26a**–**c**, Figure 15) have been tested towards human tumor cell lines (MCF-7, PC-3, A-549, HCT-116, and Jurcat) to evaluate the effect of the halide ions on the anticancer activity. All derivatives demonstrated antiproliferative activity in a low micromolar range with cytotoxicity following the order **26b** > **26a** > **26c** [161].

Caffeine was also used for the synthesis of a series of complexes with Fe(II), Co(II), Mn(II), Cd(II), Zn(II), Cu(II) and Ni(II). Studies of molecular docking showed that Zn(II) derivative **27** (Figure 15) has a good affinity with the receptor PI3Kg, a class of phosphatidylinositol 3-kinase (PI3K) implicated in several cellular processes related to cancer initiation and progression [162].

**Figure 16:**
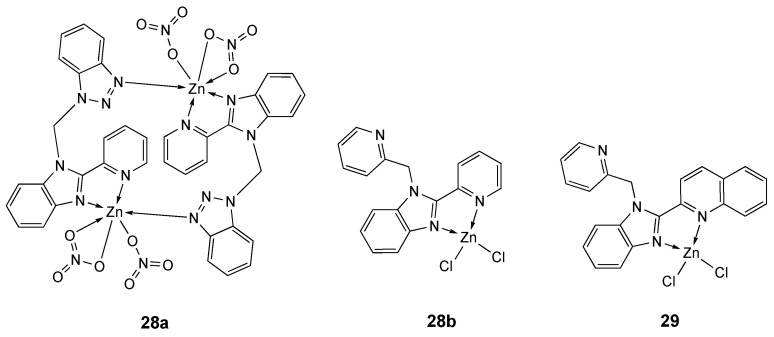
Structure of the zinc complexes **28a**, **28b** and **29**.

Two zinc complexes bearing benzimidazole-based derivatives, **28a** and **28b** (Figure 16), were structurally characterized. Compound **28a** is a dimeric complex with each Zn(II) core displaying a distorted octahedral geometry, whereas compound **28b** is mononuclear with a four-coordinated zinc center in a slightly distorted tetrahedral geometry. The metal is coordinated to two nitrogen atoms from one **L_28b_** ligand and to two chloride ions. The cytotoxic assay on several cancer cells (MCF-7, QBC939, SH-SY5Y and EC-109) showed the free ligands inactive, and complex **28a** more active than complex **28b** (at 72 h IC_50_ values of 33.0 ± 1.8, 37.2 ± 2.0, 30.3 ± 1.6 µM and 36.3 ± 2.7, and 66.6 ± 5.0, 60.1 ± 4.8, 95.7 ± 5.8 and 75.5 ± 5.2 µM, respectively, Table 8). Further studies with complex **28a** evidenced its ability to intercalate with CT-DNA and to induce morphological changes, membrane permeability increase and growth of cells in the G0/G1 phase, typical of induction of apoptosis [163].

The pyridine-benzimidazole-quinolinyl ligand **L_29_** [164,165,166] was utilized for the synthesis of Cu, Zn and Co complexes which were screened against four different esophageal cancer cell lines (SMMC-7721, BGC823, HCT-116 and HT-29) [167]. The tetrahedral zinc derivative **29** (Figure 16) showed moderate antitumor activity (average IC_50_ 57.25 µM at 72 h) (Table 9), differently from the most promising copper derivative (average IC_50_ 18.91 µM at 72 h) which was the object of more detailed studies.

**Figure 17:**
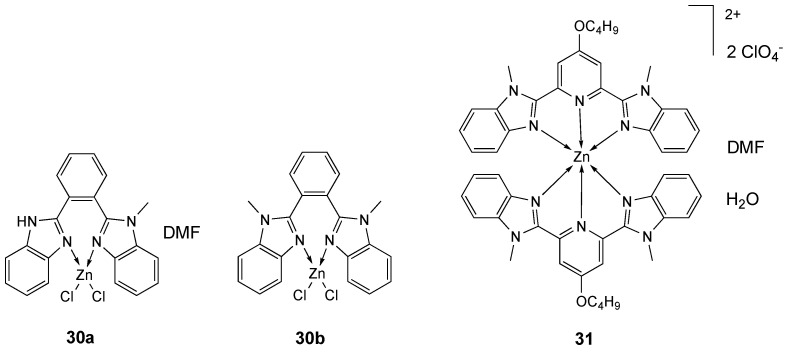
Structure of the zinc complexes **30a**, **30b** and **31**.

Bidentate benzimidazole ligands **L_30a__,b_** [168,169,170] allowed the formation of structurally characterized **30a** and **30b** (Figure 17), in which each Zn^2+^ core is tetrahedrally coordinated to two N and two Cl atoms. The cytotoxicity against MB-MDA-231 cells of Zn complexes, free ligands and cisplatin was investigated by CCK-8 assay (Table 9). The free ligands and the related complexes exhibited lower cytotoxic activities compared to the reference drug cisplatin (IC_50_ = 9.92 µM). The better cytotoxicity of complex **30b** (IC_50_ = 38.65 µM) compared to that of complex **30a** (IC_50_ > 50 µM) could be related to its higher lipophilicity (log *p* values for complexes **30a** and **30b** are 0.74 and 1.84, respectively) [171].

Tridentate benzimidazole derivative 4-butyloxy-2,6-bis(1-methyl-2-benzimidazolyl)pyridine (**L_31_**) was utilized for the synthesis of the six-coordinated **31** complex with a distorted octahedral configuration. Whereas free **L_31_** ligand has no antitumor activity against EC-109 cancer cell, Zn complex **31** (Figure 17) has a good activity (IC_50_ = 46.13 µM) (Table 9), comparable to that of cisplatin (IC_50_ = 43.99 µM), better than the analogous Co(II) derivative (IC_50_ = 75.46 µM), but lower than Cu(II) derivative (IC_50_ = 26.09 µM). The redox properties of Cu(II) and Co(II) complexes were examined by cyclic voltammetry and results exhibited irreversible redox processes. These results well evidence the importance of the nature of the metal for the biological activity [172].

## 7. Schiff Base Systems

Schiff base metal complexes have been extensively investigated due to their modular easy synthesis and versatility [173] and show interesting pharmacological properties such as anticancer [174], antibacterial [175,176] and urease inhibitory [177,178] activities. In addition, interaction of these compounds with DNA has been established [179,180] and produces different effects, including DNA molecule cleavage [181] and DNA duplex cross-linking [182].

Schiff base compounds are expected to exhibit biological properties [174,183,184,185,186,187,188] and their coordination complexes cobalt [174,189,190,191,192], copper [174,193,194,195,196] and zinc [174,196,197,198,199] acceptors are promising pharmacologically active metal compounds.

Cytotoxic activities of metal complexes derived from Schiff bases against various malignant tumors have been extensively studied and the metal which is incorporated in the complex has a great impact on the effectiveness of the compounds [200].

### 7.1. κ^2^N,N′ Systems

**Figure 18:**
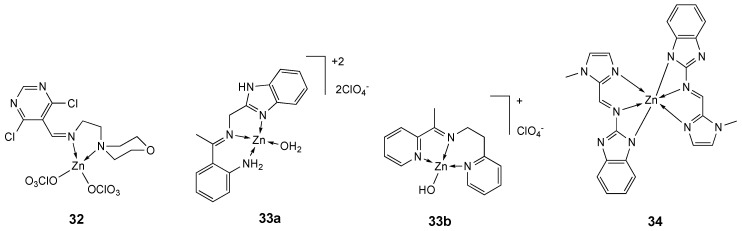
Structure of the zinc complexes **32**, **33a**, **33b** and **34**.

The Zn(II) complex **32** (Figure 18) has been prepared starting from the Schiff base ligand **L_30_**, synthesized from 4,6-dichloropyrimidine-5-carboxaldehyde and 4-(2-aminoethyl)morpholine [201]. The interaction of this complex with calf thymus (CT) DNA has been investigated by electronic absorption, fluorometric, viscometric and cyclic voltametric measurements. In vitro anticancer activity of **L_32_**, **32** and the analogous Cu(II) complex against selected cancer cell lines (A549, HepG2, HeLa) and a normal cell line (NHDF) was assessed by MTT assay. The results suggest that complex **32** has reasonable anticancer ability against tumor cell lines, showing higher IC_50_ values (A549 = 79.42 µg/mL; HepG2 = 85.39 µg/mL and HeLa = 82.39 µg/mL) on cancer cell lines than those exhibited by ligand **L_30_** (A549 = 105.15 µg/mL; HepG2 = 106.8 µg/mL and HeLa = 108.8 µg/mL) (Table 10), but lower than those of the analogous Cu(II) complex [201].

### 7.2. κ^3^N,N′,N″ Systems

The high flexibility and coordinating properties make tridentate Schiff-base ligands very fascinating and those involving oxygen and nitrogen donor ligands have generated interest in catalysis and bio-inorganic systems [176], as cleavage agents for DNA, for novel potential DNA-targeted antitumor drugs and cancer chemotherapeutic agents [64].

Two Schiff bases (ambaf = 2-[*N*-(1*H*-benzimidazol-2-ylmethyl)ethanimidoyl]aniline and apyepy = 2-(pyridin-2-yl)-*N*-[1-(pyridin-2-yl)ethylidene]-ethanamine) zinc(II) complexes (**33a**, **33b**, Figure 18) were synthesized by Vieria and co-workers and their interaction with CT-DNA was investigated by circular dichroism and UV/Vis spectroscopies. Both compounds are able to interact with DNA, electrostatically with the DNA phosphate groups or via intercalation between the base pairs. Their antiproliferative activity was investigated against human sarcoma cancer cells (MES-SA and MES-SA/DX5), in comparison to non-tumorigenic fibroblasts P4. Complex [Zn(apyepy)OH]^+^ (**33b**) was found to be non-cytotoxic (IC_50_ > 140 µM), while complex [Zn(ambaf)H_2_O]^2+^ (**33a**) resulted toxic toward all the tested cells, including noncancerous ones, showing a moderate toxicity in the range of 47 to 71 µM (Table 10). Compound **33a** was even more toxic than the analogous copper(II) complex, [Cu(ambaf)H_2_O]^2+^, suggesting that the higher cytotoxicity of the Zn(II) compound can perhaps be ascribed to its photochemical properties, a significant increase in its fluorescence being observed by interaction with calf thymus-DNA. The order of antiproliferative action cytotoxicity is in good correlation with the cellular metal uptake, probably being dependent on the ability of the complexes to enter the cells [202].

A new zinc(II) complex **34** (Figure 18), with two benzimidazole-derived ligands has been synthesized and its interaction with the human serum albumin and DNA was investigated, showing significant binding propensity. The nuclease activity of **34** was analyzed for pBR322 DNA, confirming its potential to cleave DNA. Furthermore, the cytotoxicity of the ligand and the zinc(II) complex was investigated on a panel of selected human cancer cells (HepG2, SK-MEL-1, HT018, HeLa and MDA-MB-231), showing IC_50_ values (Table 10) higher than the standard drug cisplatin and then the related copper(II) complex. Furthermore, the in vivo chronic toxicity profile of complex **34** was also studied on all of the major organs of the mice, with low toxicity results [203].

### 7.3. κ^4^N,N′,N″,N‴ Systems

**Figure 19:**
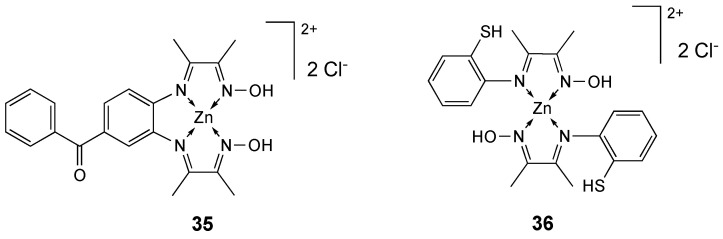
Structure of the zinc complexes **35** and **36**.

The zinc(II) complex **35** (Figure 19) of the new tetradentate Schiff base ligand **L_33_**, obtained via condensation reaction of 3,4-diaminobenzophenone with diacetyl monoxime, was successfully synthesized and its potential anticancer activity against MCF-7 cell line was investigated through MTT test: it reduced the viable cell numbers to 11% of the control samples after 72 h exposure (IC_50_ = 66 µM). Complex **35** was evaluated as a radical scavenger against 1,1-diphenyl-2-picrylhydrazyl radicals, demonstrating limited in vitro antioxidant activity in comparison with ascorbic acid [204].

The analogous Zn(II) complex **36** (Figure 19) of the ligand (2-iminothiophenol-2,3-butanedione monoxime) was synthesized and its in vitro antioxidant activity as a radical scavenger versus 1,1-diphenyl-2-picrylhydrazyl radicals was investigated (IC_50_ = 72 mg L^−1^). The binding of the complex with human serum albumin (HAS) as the model protein was examined, revealing a modest binding affinity [205].

Among N-donor ligands, pyridyl-based compounds have been deeply studied in coordination chemistry due to their strong chelating property and the ability to construct several coordination architectures [206].

**Figure 20:**
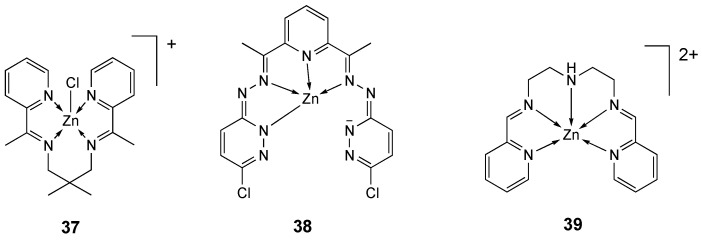
Structure of the zinc complexes **37**–**39**.

The mononuclear Zn(II) complex **37** (Figure 20) was prepared starting from the tetradentate Schiff base ligand **L_35_** and was structurally investigated by single crystal X-ray crystallography [207]. Its anticancer activity against human breast adenocarcinoma cell line was examined, showing a reduction in MCF-7 cell line viability, with increasing concentration of zinc complex after 24 h of exposure. The LC_50_ value for **37** was 12 μg/mL, suggesting an interesting cellular toxicity over MCF-7 cell line, generally induced by the endocytosis and release of ions, which promote ROS generation [207].

The Schiff base-type ligand **L_38_** [208] and its complexes with Co(III), Ni(II), Cu(II) and Zn(II) (**38**) metal centers were synthesized and their structural and physicochemical properties investigated by density functional theory (DFT) [209]. The biological activity of **L_38_** and its coordination compounds was studied on antiproliferative effects, cytotoxic effects and inhibitory effect of the ATP-binding cassette (ABC) transporter P-glycoprotein encoded by human MDR1 gene on L5178Y tumor cells. The cobalt(III) compound was the most effective inhibitor of the ABC transporter PGP drug efflux pump that is responsible for extruding the anticancer drugs from cancer cells in in vitro studies. The zinc(II) complex **38** (Figure 20) showed about half the effect when compared to analogous Co(III) complexes, whereas the compounds with Ni(II) and Cu(II) were practically inactive [209].

### 7.4. κ^5^N,N′,N″,N‴,N⁗ Systems

Adam and co-workers [210] proposed the use of the membrane-penetrating peptide Novicidin (NVC) [211] conjugated with the Schiff base-zinc complex **39** (Figure 20) as a carrier vehicle for the delivery of zinc to human prostate cancer cells. Molecular analyses were used to confirm the activation of zinc stress (e.g., ZnT-1) and apoptosis (e.g., CASP-1) genes. The cytotoxicity of the **36**-NVC complex was examined in human prostate cell lines PC3 and PNT1A, using the MTT assay. NVC alone reduced cell viability by 50% at a concentration of 16 nM and by 100% at 63 nM after 24, in both PC3 and PNT1A cell lines. The **39** complex without peptide also displayed meaningful toxicity toward both cell lines compared with NVC alone, reducing cell viability by 40% at 125 nM and by 100% at 250 nM after 24 h. **39**-NVC complex reduced cell viability in PC3 cell line by 65% at a concentration of 46 nM, but in PNT1A cells it showed negligible cytotoxicity, even at a concentration of 500 nM [210]. Zinc uptake was confirmed in both cell lines.

## 8. Miscellanea Systems

**Figure 21:**
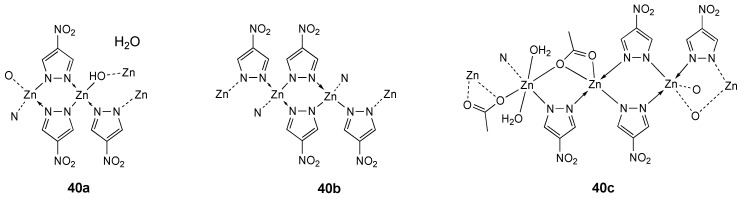
Structure of the zinc complexes **40a**–**c**.

A series of metal-organic chains (MOCs) based on 4-nitro-1*H*-pyrazole as ligand and zinc as metal center has been reported and structurally characterized [212]. The antitumor properties of the 4-nitro-1*H*-pyrazole ligand (**HL_40_**) and the MOCs complexes **40a** ({[Zn_2_(μ-4-NO_2_-pz)_3_(μ-OH)]·H_2_O}_n_), **40b** ({[Zn_2_(μ-4-NO_2_-pz)_4_]}_n_) and **40c** ({[Zn_3_(μ-4-NO_2_-pz)_4_(μ-ac)_2_(H_2_O)_2_]}_n_) (Figure 21) were evaluated against three cancer cell lines (HT29, Hep-G2 and B16-F10). The sTable 1D Zn-coordination complexes **40b** and **40c** showed a similar antitumor activity (average IC_50_ 48.23 and 45.13 μg/mL for **40b** and **40c** respectively), lower IC_50_ than the free ligand (average IC_50_ 136.67 μg/mL), and with low specificity with respect to cell type (Table 11). MOCs complexes **40b** and **40c**, prepared using water as solvent, can avoid the potential self-aggregation issue often encountered by some antitumor compounds such as triterpenes and minimize generic interactions, representing an alternative to traditional coordination complexes [212].

**Figure 22:**
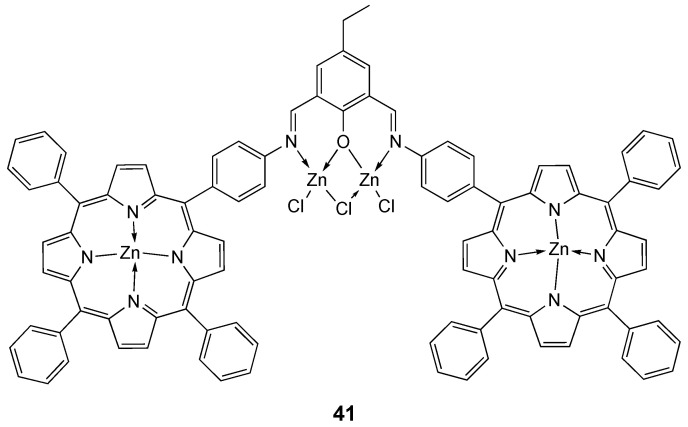
Structure of the zinc complex **41**.

A new porphyrin-Schiff base ligand and its Zn(II) complex **41** (Figure 22) were synthesized by Tümer and co-workers. Superoxide dismutase activities of **41** were investigated in comparison with analogous Cu(II), Fe(III), Mn(III) and Pt(II) complexes. Additionally, the DNA (fish sperm FSdsDNA) binding studies of the complex was performed by UV-vis spectroscopy (K_b_ = 1.3 × 10^6^). Competitive studies with ethidium bromide (K_b_ = 1.23 ± 0.07 × 10^5^) showed that the compounds interact efficiently with DNA through an intercalating way [213].

**Figure 23:**
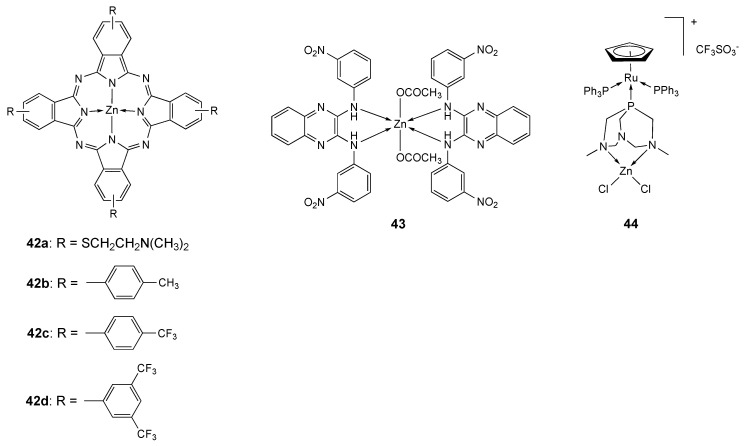
Structure of the zinc complexes **42**–**44**.

Phthalocyanines are natural aromatic and planar macrocycles with a structure similar to porphyrins. Lipophilic phthalocyanines (Pcs) [214,215] incorporated into the poloxamine Tetronic^®^ 1107 (T1107), an amphiphilic poly(ethylene oxide)-poly(propylene oxide) block copolymer containing two tertiary amine groups, resulted highly efficient against different human and murine colon tumor cell lines (IC_50_ = ∼10 nM) after irradiation [216]. Besides, **42a**-T1107 (Figure 23) induced an apoptotic cell death both in two- and three-dimensional colon carcinoma cell cultures [217]. In addition, the in vivo effect of photodynamic therapy (PDT) with **42a**-T1107 in a CT26 murine colon carcinoma model was explored [218]. **42a**-T1107 inhibited tumor growth and prolonged mice survival, without signs of tissue-specific or systemic toxicity, inducing an apoptotic tumor cell death [218].

A series of symmetrically tetra-substituted thiophenyl zinc(II)phthalocyanines **42b**–**d** (Figure 23) was reported and their antiproliferative activity was tested against A549, MCF-7 and HepG2 tumor cell lines and for comparison against healthy normal cells (human fibroblast cells). Compound **42d**, containing eight CF_3_ groups attached at positions 3,5 to the phenyl ring in its phthalocyanine scaffold, demonstrated to be the most potent of the series with a good selectivity towards cancer cells compared to healthy cells. The IC_50_ values obtained for **42d** against MCF7, HepG2 and A549 cell lines are 3.75, 3.27 and 6.03 µM respectively. No PDT applications of compounds **42b**–**d** have been reported [219].

The octahedral complex **43** (Figure 23) comprising the ligand *N*^2^,*N*^3^-bis(3-nitrophenyl)quinoxaline-2.3-diamine (**L_43_**) [220] was tested against HeLa cell line together with analogous Co, Ni and Cu derivatives [221]. Complex **43** exhibited the highest activity (IC_50_ = 35.29 µM, Table 12) in comparison to the free ligand (IC_50_ > 100 µM) and to the other metal complexes (IC_50_ 132.50, 65.09 and 65.62 μM for Co, Ni and Cu derivative respectively). Moreover, Compound **43** showed both DNA binding through intercalation and effective DNA cleavage. Molecular docking study against human papilloma virus (HPV) receptor molecule and the ATP binding site of telomerase showed that **43** is more potent against HPV receptor.

Heterobimetallic complexes are attracting a wide interest as potential metal-based drugs and theranostic agents. Romerosa et al. following previous studies on Ru-Co [223] derivatives and on Ru-complexes comprising the neutral ligand 3,7-dimethyl-1,3,7-triaza-5-phosphabicyclo[3.3.1]nonane (dmoPTA) [224,225,226,227,228,229,230], able to coordinate different metals through the soft P and the two hard NCH_3_ groups, reported the synthesis and characterization of **44** (Figure 23) [222]. The Zn atom is coordinated to two N-atoms of the dmoPTA ligand and to two chloride ions in a tetrahedral environment, whereas Ru is coordinated to three P atoms (two from PPh_3_ molecules and one from bridging dmoPTA). Heterobimetallic Ru-Zn derivative **44** was found to be very stable in a mixture of [D_6_]DMSO/cell-culture medium (t = 48 h) and its antiproliferative activity was tested on six human solid tumor cells lines (A549,HBL-100, HeLa, SW1573, T-47D and WiDr) and against human fibroblast (non-tumor) cell line BJ-hTert (Table 12). It displayed antitumor activity higher than the Ru-Co analogous complex (1.2–2.5 times) and cisplatin (26–426 times), with GI_50_ values in the range 0.030–0.083 µM. Moreover its activity against tumor cell lines was 3–8 times higher than against non-tumor cell line, indicating a good selectivity [222].

## 9. Concluding Remarks

Notwithstanding our choice to survey only Zn complexes containing N-donor ligands, the high number of papers published in this restricted field in the last five years evidences the high vitality of the research on this topic. We have surveyed 100 complexes and the majority of them displayed some antiproliferative activity in vitro. Unfortunately, literature data are not always comparable as IC_50_ values are calculated at different incubation time (24, 48, or 72 h) and reported with different units. Moreover, zinc complexes have been synthesized for comparison to analogous complexes of other transition metals, M(II), which often resulted more active so that their biological behavior, not that of zinc derivatives, has been deeply studied. In the Supplementary Materials Section we have reported the studies performed to determine the mechanism of action of the complexes and the main outcomes (Table S1, Supplementary Materials).

In order to find out a possible correlation between the cytotoxic activity in vitro and the chemical features of the surveyed complexes, we have cumulated the most active reported species (43 out of 102, i.e., all the complexes which exhibit an antitumoral activity with IC_50_ values ≤ 10 µM (Table 13 against one or more cancer cell lines, reaching in some cases the nanomolar range. In detail, penta-coordinated terpyridine derivatives **14a**–**j**, [141] **15a**–**h** [136] and **16** [142] and hexacoordinate bis-terpyridine **17** exhibited very high activity towards a large panel of cancer cell lines, and in the case of **16** and **17** demonstrated also a good specificity for tumor cells. On the other hand, at least for **16** and **17**, the free ligand is highly active per se and metal coordination did not lead to any improvements. As it can be seen in the table, this behavior can be observed also with other complexes, such as **8**, **12** and **13**. In these classes of compounds, zinc coordination of active ligands did not determine an evident synergic effect, but the activity of the final complexes was comparable to that of the free ligands.

Concerning the geometry of the most active complexes, six coordination is the predominant mode even though in many cases the coordination sphere is filled with one or two water molecules (i.e., compounds **8**, **11**, **12**, **13**, **20**, **21**), even if given the high labile character of Zn(II) complexes and stereochemical non-rigidity, in solution or even in cell medium it is very probable to observe changes to higher C.N. or even further speciations. It’s worth noting that the design of binuclear, homo- and hetero-metallic derivates led to very active species such as **2**, **10** and **44**.

Probably due to the fact that DNA is considered the major target for Zn derivatives, anticancer activity has been rarely tested against Pt-resistant cancer cell lines. Interestingly, when reported, cytotoxicity data towards healthy cell lines showed a good selectivity for tumoral cell line with SI values up to 30. Anyway, notwithstanding the promising IC_50_ and SI values, none of the complexes reported in Table 13 were validated in in vivo tests. In our survey, only 2 papers [101,218] report some in vivo data. In particular, complex **5a**, showing high antitumor activity against HepG2 and SMMC-7721 cell lines and low toxicity in ICR mice, might represent a potential new approach for the treatment of hepatocellular carcinoma. Concerning the phthalocyanine **42a** incorporated into T1107, its ability to reduce the tumor growth of PDT treated colon carcinoma in mice without inducing systemic and tissue toxicity makes it a potential clinical candidate for the treatment of colorectal cancer.

Considering that antiproliferative activity in vitro is not predictive of an activity in vivo, it would be desirable an effort to perform in vivo experiments with the most promising candidates to effectively evaluate the potential of Zn-based anticancer agents.

In addition to the complexes showing micromolar or sub-micromolar anticancer activity, a handful of compounds (**1a**, **4b**, **5a**, **7**, **9**, **18a**, **19a**, **19b**, **24b**, **34**) exhibited a noticeable antitumor activity with IC_50_ values of 10–20 µM, whereas the remaining ones showed a moderate/low activity.

The surveyed complexes represent a fraction out of all coordination zinc complexes tested as antitumor agents, i.e., only complexes containing N-donor ligands, which, anyway, include many important classes of ligands such as terpyridine, diimine, Schiff bases, pyrazolates and so on. Looking critically at these data, in particular the cytotoxic activity in vitro, without making any considerations on action mechanisms and proposed targets, we can summarize some general findings that could be useful for the future research in this field. As far as the chemical structure is concerned, among the different geometries which Zn can adopt, hexa- and penta-coordination are by far the most common situations for active compounds differently from zinc proteins where tetrahedral coordination, frequently slightly distorted, is the preferred geometry. The hapticity of the ligands is not decisive for the activity of the final complex, whereas the frequent presence of water molecules in the coordination sphere can allow an easy exchange with biological substrates. The use of active ligands does not always determine an increase of cytotoxicity upon coordination. From studies carried on with analogous complexes of different bivalent metal (such as Ni(II), Cu(II), Co(II), Fe(II), Mn(II)), it very often came out that zinc derivatives were less active, suggesting a minor effect of the metal compared to other metals.

As reported in the Introduction, the development of a zinc- based strategy against cancer can have two opposite approaches: (i) chelation therapy for zinc removal or (ii) use of zinc derivatives to increase its concentration in the tumor cells. In the latter case, the aim can be merely to restore Zn deficiency by using ionophore systems or, as in the case of the surveyed coordination complexes, to exert an antitumor activity acting on specific targets. From the above considerations, it seems that, excluding the use of hypotoxic Zn as a carrier of photoactive species for PDT or of active ligands, the antitumor efficacy of Zn-coordination complexes is not so appealing in comparison to other metal-based derivatives. The concentration of Zn in cells is probably so (relatively) high, that small variations do not induce an antiproliferative action of the metal unless specific mechanisms are involved. The reported studies have been mainly focused on DNA or HAS interactions, even though TOPO-I-II, p53, nuclease have been proposed as alternative targets. In our opinion, to obtain more performants agents more detailed studies on potential targets should be pursued. Another aspect, which should be evaluated, is the possibility to use the low active, but at the same time low toxic zinc derivatives in combination with other chemotherapeutic agents to reduce their side effects.

An in-depth analysis concerning all the classes of zinc coordination complexes, not only those with N-donor ligands, is in progress to confirm the above general considerations.

## Figures and Tables

**Table 1:** IC_50_ values (µM) for zinc complexes **1a**, **1b** and **2a**, **2b**, ligands **L_1a_** and **L_1b_** and the reference compounds cisplatin, ZnCl_2_ and Zn(ClO_4_)_2_ against different human tumor and normal (HL-7702) cell lines, after an incubation time of 48 h.

Compound [Ref.]	Cell Line						
	BEL-7404	SK-OV-3	A-549	A-375	MGC-803	NCI-H460	HL-7702
**1a** [74]	13.24	17.23	ND	24.38	15.66	34.54	87.78
**1b** [74]	3.26	7.31	13.21	8.79	6.32	11.89	49.65
**L_1a_** [74]	38.41	67.58	31.55	37.67	55.41	>100	>100
**L_1b_** [74]	27.57	56.32	54.61	28.15	38.26	88.45	>100
**2a** [75]	6.69	7.33	>100	28.30	4.66	16.51	52.32
**2b** [75]	2.07	9.27	26.11	11.62	0.72	14.82	39.60
Cisplatin [74]	64.22	84.21	17.21	78.54	23.58	48.52	74.25
ZnCl_2_·6H_2_O [75]	73.54	ND ^a^	88.43	ND ^a^	58.31	36.14	>100
Zn(ClO_4_)_2_·6H_2_O [74]	74.21	ND ^a^	97.44	ND ^a^	53.28	64.10	>100

^a^ No data available.

**Table 2:** IC_50_ values (µM) for zinc complexes **4**–**7**, free ligands phen, ida, bpy and Hnor and the reference compounds cisplatin, ZnCl_2_ and ZnSO_4_ against different tumor and normal (LO_2_ and PBMC) cell lines, after an incubation time of 48 h.

Compound [Ref.]	Cell Line								
	MCF-7	HeLa	KB	LO_2_	HepG2	SMMC-7221	A2780	DL	PBMC
**3** [99]	100 ^a^								
**4a [100]**		23.5	25.6	30.1					
**4b** [100]		12.4	15.2	16.3					
**5a** [101]					10.01	11.75			
**5b** [101]					41.63	44.36			
**5c** [101]					22.34	27.02			
**5b** + **5c** (1:1) [101]					13.75	12.58			
Phen [101]					68.31	61.8			
Ida [101]					52.72	49.14			
**6** [102]							1.26 ^a^		
6 + CuCl_2_ [102]							1.23 ^a^		
Phen [102]							3.70 ^a^		
Bpy [102]							10.70 ^a^		
Hnor [102]							<200 ^a^		
**7** [103]								17.12 ^b^	63.23 ^b^
Cisplatin		11.9 [100]	13.8 [100]	9.8 [100]			1.9 ^a^ [102]	0.45 [103]	6.31 [103]
ZnCl_2_ [101]					>200	>200			
ZnSO_4_ [101]					>200	>200			

^a^ Incubation time of 72 h, ^b^ Incubation time of 24 h.

**Table 3:** IC_50_ values (µM) for zinc complexes **8**–**13**, ligands and the reference compounds cisplatin and doxorubicin against different tumor and normal (V79) cell lines after an incubation time of 48 h.

Compound [Ref]	Cell Line						
	A2780	MCF-7	HeLa	V79	A529	HCT113	MDA-MB-231
**8a** [113]	5.8	17.6	26.9	23.9			
**8b** [113]	25.9	21.8	37.7	28.1			
**8c** [113]	3.40	12.3	15.9	10.8			
**8d** [113]	5.6	18.2	23.7	33.6			
**8e** [113]	1.73	3.04	4.58	4.06			
**8f** [113]	2.4	9.10	16.6	14.2			
**8g** [113]	0.75	5.42	8.16	6.78			
Phen [113]	5.84	6.21	11.8	7.10			
Clphen [113]	5.70	10.0	12.0	9.50			
Amphen [113]	1.84	4.41	7.20	3.90			
Epoxyphen [113]	14.4	10.0	18.4	18.7			
Bphen [113]	0.50	3.20	2.00	1.20			
**9** [114]		11.0 ^a^				31.2 ^a^	
**L_9_** [114]		41.1 ^a^				29.6 ^a^	
2,2′-Bipyridine [114]		12.0 ^a^				20.0 ^a^	
**10** [115]			2.13 ^a^		1.37 ^a^		
**11** [116]		0.165 (LC_50_) ^a^ 0.016 (GI_50_) ^a^					
**12** [117]							0.5
**13** [117]							0.4
Phendione [117]							0.3
Phendione + HNPR [117]							0.4
Phendione + HMFN [117]							0.4
Cisplatin [113]	22.5	20.7	3.59	23.5	1.0		
Doxorubicin [116]		0.183(LC_50_) ^a^ 0.018 (GI_50_) ^a^					

^a^ Incubation time of 72 h.

**Table 4:** IC_50_ values (µM) for terpyridine complexes **14**–**19**, the free ligand **L_16_** and the reference compounds cisplatin, oxaliplatin and doxorubicin against different tumor and normal (NHDF and MRC-5) cell lines, after an incubation time of 72 h.

Compound [Ref]	Cell Lines												
	A-549	Bel-7402	MCF-7	Eca-109	PANC-1	HCT-116	U-251	NHDF	HCT-116	MRC-5	RL952	MDA-MB-231	HeLa
**14a** [141]	0.440	1.309	1.486	1.251									
**14b** [141]	0.933	1.842	2.769	2.017									
**14c** [141]	0.756	1.470	1.358	1.821									
**14d** [141]	1.042	1.883	0.589	3.320									
**14e** [141]	0.586	1.435	1.187	1.722									
**14f** [141]	0.435	0.660	1.956	1.198									
**14g** [141]	0.633	1.636	0.374	1.045									
**14h** [141]	1.228	1.557	2.428	1.215									
**14i** [141]	1.270	1.804	3.548	1.280									
**14j** [141]	0.333	0.730	1.764	1.193									
**15a** [136]	0.094	0.055	0.244										
**15b** [136]	0.059	0.069	0.440										
**15c** [136]	0.076	0.089	0.336										
**15d** [136]	0.147	0.604	0.788										
**15e** [136]	0.121	0.106	0.642										
**15f** [136]	0.149	0.741	0.899										
**15g** [136]	0.155	0.507	1.311										
**15h** [136]	0.141	0.656	0.917										
**16** [142]	6.31		0.22		0.59	3.72	2.23	>25					
**L_16_** [142]	0.75		0.04		0.44	0.27	0.14	20.83					
**17** [142]	1.56		0.15		0.13	0.98	0.72	14.19					
**18a** [143]									10.0 ^b^	94.0		23.0	
**18b** [143]									149.7	87.7		154.5	
**19a** [143]			12.58 ^a^								33.63 ^a^		18.47 ^a^
**19b** [143]			27.45 ^a^								11.71 ^a^		15.57 ^a^
Cisplatin	5.082 [141] 3.986 [136]	3.088 [141] 3.088 [136]	11.49 [141] 5.143 [136] 26.43 ^a^ [143]	11.99 [141]									10.08 ^a^ [143]
Doxorubicin [142]	1.06		0.41		0.73	0.34	0.05	0.14					
Oxaliplatin [142]	>25		1.13		>25	2.23	2.16	>25					

^a^ Incubation time of 48 h; ^b^ Incubation time of 24 h.

**Table 5:** IC_50_ values (µM) for zinc complexes **20**, **21a**,**b** and reference cisplatin against HeLa and KB tumor cell lines after an incubation time of 24, 48 or 72 h.

Compound [Ref.]	Cell Lines	
	HeLa	KB
**20** [147]	6.48 (24 h) 4.95 (48 h)	8.98 (24 h) 6.48 (48 h)
**21a** [148]	18.63 (24 h) 2.56 (72 h)	
**21b** [148]	13.24 (24 h) 1.43 (72 h)	
Cisplatin	4.38 (24 h) [147] 3.21 (48 h) [147] 15.36 (24 h) [148] 1.97 (72 h) [148]	6.23 (24 h) [147] 4.78 (48 h) [147]

**Table 6:** IC_50_ values (µM) for zinc complexes **22a**, **22b and 23c**, **23d** against different tumor cell lines after an incubation time of 72 h.

Compound [Ref]	Cell Lines					
	SMMC-7721	HT-29	MCF-7	MDA-MB-231	K562	HL-60
**22a** [149]	3.98					
**22b** [149]	9.78					
**23c** [155]		NA ^a^	NA ^a^	38.3	NA ^a^	NA ^a^
**23d** [155]		13	NA ^a^	NA ^a^	NA ^a^	NA ^a^

^a^ IC_50_ values were not determined as the compounds were not found to be significantly active at 100 mM.

**Table 7:** IC_50_ values (µM) for zinc complexes **24** and **25**, and references cisplatin and docetaxel against different tumor and normal (BEAS.2B) cell lines after an incubation time of 72 h.

Compound [Ref]	Cell Line			
	A549	BEAS-2B	A2780	DU-145
**24a** [159]	1.97	59.8		
**24b** [159]	13.77	61.59		
**24c** [159]	1.9	32.67		
**24d** [159]	9.36	51.55		
**24e** [159]	28.55	38.24		
**24f** [159]	22.36	60.04		
**24g** [159]	77.46	>100		
**25a** [160]			0.11	6.92
**25b** [160]			0.05	67.6
**25c** [160]			0.60	141.3
**25d** [160]			0.69	11.5
**25e** [160]			0.19	79.4
**25f** [160]			33.1	>1000
**25g** [160]			2.57	>1000
**25h** [160]			17.4	208.9
**25i** [160]			151.4	93.3
Cisplatin [159]	2.56	2.23		
Docetaxel [160]			0.15	0.07

**Table 8:** IC_50_ values (µM) for zinc complexes **28a**, **28b**, ligands **L_28a__,b_** and reference cisplatin against different tumor cell lines after an incubation time of 72 h.

Compound [Ref]	Cell Lines			
	MCF-7	EC-109	SHSY5Y	QBC939
**28a** [163]	33.0	37.2	30.3	36.3
**28b** [163]	66.6	60.1	95.7	75.5
**L_28a_** [163]	125.0	90.4	88.3	85.9
**L_28b_** [163]	>150	124.6	>150	>150
Cisplatin [163]	17.5	13.3	25.3	126.9

**Table 9:** IC_50_ values (µM) for zinc complexes **29**–**31** and reference compound cisplatin against different tumor and normal (LO2) cell lines, after an incubation time 72 h.

Compound [Ref]	Cell Lines						
	SMMC7721	BGC823	HCT116	HT29	LO2	MDA-MB-231	EC-109
**29** [167]	49.9	45.5	64.8	68.8	36.6		
**30a** [171]						>50 ^a^	
**30b** [171]						38.7 ^a^	
**L_30a_** [171]						>50 ^a^	
**L_30b_** [171]						>50 ^a^	
**31** [172]							46.13
Cisplatin	8.22 [167]	8.0 [167]	40.3 [167]	47.7 [167]	6.75 [167]	9.9 [172]	43.99 [172]

^a^ Incubation time of 48 h.

**Table 10:** IC_50_ values (µM) for zinc complexes **32**–**34**, ligands **L_32_** and **L_34_** and reference compound cisplatin against different tumor and normal (NHDF and P4) cell lines, after an incubation time 72 h.

Compound [Ref]	Cell Lines									
	A549	HepG2	HeLa	NHDF	MES-SA	MES-SA/Dx5	P4	SK-MEL-1	HT018	MDA-MB 231
**32** [201]	79.4	85.4	82.4	109.2						
**L_32_** [201]	105.2	106.8	108.8	109.5						
**33a** [202]					47.0	71.2	54.5			
**33b** [202]					>140	>140	>140			
**34** [203]		19	24.5					18	25	26.7
**L_34_** [203]		NA	NA					NA	NA	NA
Cisplatin [203]		6	6					5.6	5.7	3.1

**Table 11:** IC_50_ values (µg/mL) for zinc complexes **40b** and **40c** and ligand **HL_40_** against different cancer cell lines, after an incubation time of 72 h.

Compound [Ref]	Cell Lines		
	HT29	Hep-G2	B16-F10
**40b** [212]	46.7	45.4	52.6
**40c** [212]	41.8	45.8	47.8
**HL_40_** [212]	97.6	210.6	101.8

**Table 12:** IC_50_ values (µM) for zinc complex **42b**–**d**, **43** and **44** against different tumor and normal (Bj-hTert) cell lines, after an incubation time of 48 h.

Compound [Ref]	Cell Lines								
	A549	HBL-100	HeLa	SW1573	T-47D	WiDr	BJ-hTert	MCF-7	HepG2
**42b** [219]	25.4							5.30	5.28
**42c** [219]	31.8							4.60	21.68
**42d** [219]	6.03							3.75	3.27
**43** [221]			35.29 ^a^						
**44** [222]	0.036 ^b^	0.072 ^b^	0.051 ^b^	0.030 ^b^	0.083 ^b^	0.054 ^b^	0.023 ^b^		
Cisplatin [222]	4.9 ^b^	2.9 ^b^	1.8 ^b^	2.7 ^b^	17 ^b^	23 ^b^	14 ^b^		

^a^ Incubation time of 72 h; ^b^ GI_50_ values (µM).

**Table 13:** Zinc compounds which showed an antiproliferative activity characterized by IC_50_ values ≤ 10 µM.

Compound	Coord. Number	Tumor Cell Lines (IC_50_ ≤ 10 µM)	Normal Cell Lines (IC_50_ µM, SI)	[Ref.]	Ligand Activity (IC_50_ µM)	Incubation Time
**1b**	5	BEL-7404, SK-OV-3, A-375, MGC-803	HL-7702 (49.65, 7.7)	[74]	average 37.58	48 h
**2a** ^§^	5	BEL-7404, SK-OV-3, MGC-803	HL-7702 (52.32, 11.2)	[75]	average 53.8	48 h
**2b** ^§^	5	BEL-7404, SK-OV-3, MGC-803 *	HL-7702 (39.60, 9.85)	[75]	average 40.72	48 h
**6a**	6	A2780		[102]		72 h
**8a**	6	A2780	V79 (23.9, 4.1)	[113]	5.84	48 h
**8c**	6	A2780	V79 (10.8, 3.2)	[113]	1.84	48 h
**8d**	6	A2780	V79 (33.6, 6.0)	[113]	14.4	48 h
**8e**	6	A2780, MCF-7, HeLa	V79 (4.06, 2.3)	[113]	average 1.9	48 h
**8f**	6	A2780, MCF-7	V79 (14.2, 5.9)	[113]	average 6.02	48 h
**8g**	6	A2780 *, MCF-7, HeLa	V79 (6.78, 9.0)	[113]	average 4.48	48 h
**10** ^§^	5,6	A549, HeLa		[115]		72 h
**11**	6	MCF-7		[116]		48 h
**12**	6	MDA-MB-231	RAW 264.7 (2.0, 4.0)	[117]	0.4	72 h
**13**	6	MDA-MB-231	RAW 264.7 (1.7, 4.25)	[117]	0.4	72 h
**14a–j**	5	A-549 *, Bel-7402 *, MCF-7 *, Eca-109		[141]		72 h
**15a–h**	5	A-549 *, Bel-7402 *, MCF-7 *		[136]		72 h
**16**	5	A-549, MCF-7 *, PANC-1 *, HCT-116, U-251	NHDF (>25, >9.6)	[142]	average 0.33	72 h
**17**	6	A-549, MCF-7 *, PANC-1 *, HCT-116 *, U-251 *	NHDF (14.19, 20)	[142]	average 0.33	72 h
**20**	6	HeLa, KB		[147]	>50	48 h
**21a,b**	6	HeLa		[148]		72 h
**24a**	4	A-549	BEAS-2B (59.8, 30.3)	[159]		72 h
**24c**	4	A-549	BEAS-2B (32.67, 17.2)	[159]		72 h
**24d**	4	A-549	BEAS-2B (51.55, 5.5)	[159]		72 h
**42b–d**	4	MCF-7, HepG2		[219]		48 h

* sub-micromolar IC_50_ values; ^§^ binuclear.

## Supplementary Materials

The following are available online, Table S1: Studies performed to determine the mechanism of action of selected zinc(II) complexes.

## Abbreviations

ambaf	2-[*N*-(1Hbenzimidazol-2-ylmethyl)ethanimidoyl]aniline
amphen	5-amine-1,10-phenanthroline
apyepy	2-(pyridin-2-yl)-*N*-[1-(pyridin-2-yl)ethylidene]-ethanamine
Bphen	4,7-diphenyl-1,10-phenanthroline
bib	1,3-bis(imidazol-1-yl)benzene
bip	3,5-bis(1-imidazoly)pyridine
bpy	2,2-bipyridine
CT	calf thymus
cisplatin	*cis*-diamminedichloroplatinum(II)
dmoPTA	3,7-dimethyl-1,3,7-triaza-5-phosphabicyclo[3.3.1]nonane
H_2_bdc	1,4-dicarboxybenzene
H_2_tib	1,3,5-tris(1-imidazolyl)benzene
HMFN	mefenamic acid
Hnor	norharmane
HNPR	naproxen
HPV	human papilloma virus
HSA	human serum albumin
IC_50_	concentration affording 50% of inhibition
Ida	iminodiacetate
MOCs	metal-organic chains
NaNPR	sodium naproxen
NH_2_-H_2_bdc	2-amino-1,4-dicarboxybenzene
NSAID	non-steroidal anti-inflammatory drug
NVC	novocidin
Pcs	phthalocyanines
Pdc	2,6-pyridine dicarboxylate
PDT	photodynamic therapy
phen	1,10-phenantroline
phendione	1,10-phenanthroline-5,6-dione
ROS	reactive oxygen species
Sal-Gly	*N*-salicylideneglycinate
SARs	structure-activity relationships

## Acronyms of Cell Lines Cited in This Review

A2780	human ovarian carcinoma
A-375	human melanoma
A-549	human alveolar basal epithelial cancer
B16-F10	*mus musculus* skin melanoma
BEAS-2B	human lung epithelial cancer
Bel-7402	human liver carcinoma
Bel-7404	human hepatoma
Bel-7042	human hepatocellular carcinoma
BJ-hTer	human fibroblast
BGC823	human gastric cancer
CT26	murine colon carcinoma
Du-145	human prostate carcinoma
Eca-109	squamous carcinoma
HBL-100	human breast
HCT-116	human colon carcinoma
HeLa	human cervical carcinoma
Hep-G2	human hepatocellular carcinoma
HL-60	human promyelocytic leukemia
HL-7702	normal human liver
HT018	human colon cancer
HT-29	human colon carcinoma
Jurcat	human T lymphocyte
K562	human chronic myelogenous leukemia
KB	human cervix carcinoma
L5178Y	mouse T-cell lymphoma
LO_2_	human immortal hepatic cell line
MCF-7	human breast carcinoma
MDA-MB-231	human breast carcinoma
MES-SA	human uterine sarcoma
MES-SA/Dx5	multi drug-resistant cell line derived from the MES-SA
MGC-803	gastric cancer
MRC-5	normal lung tissue
NCI-H460	human non-small cell lung carcinoma
NHDF	normal human dermal fibroblasts
P4	human foreskin fibroblasts
PANC-1	human pancreatic cancer cell line
PBMC	peripheral blood mononuclear cells
PC-3	human prostatic carcinoma
PI3K	phosphatidylinositol 3-kinase
PNT1A	human immortalized prostatic cell line
QBC939	human cholangiocarcinoma (resistant to cisplatin)
RAW 264.7	mouse macrophage
RL952	human endometrial carcinoma
SH-SY5Y	human neuroblastoma
SK-MEL-1	human melanoma
SK-OV-3	human ovarian cancer cell line
SMMC-7721	human hepatocellular carcinoma
SW1573	human lung
T-47D	human breast
U-251	malignant glioblastoma
V79	Chinese hamster lung fibroblasts
WiDr	human colon carcinoma
