# Supplementary material for: Zinc Complexes with Nitrogen Donor Ligands as Anticancer Agents
Source: Molecules. 2020 Dec 9;25(24):5814. doi: 10.3390/molecules25245814 (PMC7763991; doi:10.3390/molecules25245814)
Supplement: Supplementary file 1 [file molecules-25-05814-s001.pdf]

Supporting Information

Review

Zinc Complexes with Nitrogen Donor Ligands as Anticancer Agents

Marina Porchia [1,*], Maura Pellei [2,*], Fabio Del Bello [3] and Carlo Santini [2]

[1] *ICMATE-C.N.R., Corso Stati Uniti, 4, 35127 Padova, Italy*

[2] *School of Science and Technology, Chemistry Division, University of Camerino, via S. Agostino 1, 62032 Camerino (MC), Italy*

[3] *School of Pharmacy, Medicinal Chemistry Unit, University of Camerino, Via S. Agostino 1, 62032 Camerino (MC), Italy*

* Correspondence: marina.porchia@cnr.it (Marina Porchia); maura.pellei@unicam.it (Maura Pellei)

Table S1. Studies performed to determine the mechanism of action of selected zinc(II) complexes.

Compound	Cell lines	Mechanism analysis	Method	Main outcomes
**1b**	BEL-7404	cellular uptake	plasma-mass spectrometry (ICP-MS)	noticeable cellular uptake and DNA accumulation,<br>induction of cancer cell apoptosis through mitochondrial pathways,<br>DNA interaction via an intercalating mode
		apoptosis	flow cytometry, confocal microscopy, western blot analysis, mitochondrial membrane potential detection	
		DNA binding	fluorescence studies with<br>ethidium bromide (EB), circular dichroism (CD) spectrophotometry, viscometric measurements	
**2b**	MGC-803	cellular uptake	ICP-MS	noticeable cellular uptake and DNA accumulation, induction of cancer cell apoptosis via the intrinsic pathway, DNA damage due to ROS overproduction
		apoptosis	flow cytometry, confocal microscopy, western blot analysis	
		DNA damage	comet assay	
		ROS generation	DCFH-DA fluorescence analysis	
**3**		DNA binding	UV-vis titration experiments, fluorescence studies with EB, CD spectrophotometry molecular docking studies	DNA interaction via groove binding propensity, DNA cleavage via hydrolytic pathway
		DNA cleavage	agarose-gel electrophoresis experiments	
**4a-b**	HeLa, KB	apoptosis	flow cytometry, confocal microscopy	DNA interaction with different binding affinity (**7b** > **7a**)<br>DNA-cleavage activity, induction of cancer cell apoptosis
		DNA binding	UV-vis titration experiments, fluorescence studies with EB, molecular docking studies	
		DNA cleavage	agarose-gel electrophoresis experiments	
**5a**	HepG2, SMMC-7721	cell cycle analysis	flow cytometry	cell cycle arrest at G0/G1 phase.
**7**	DL	apoptosis	flow cytometry, confocal microscopy	induction of cancer cell apoptosis, interaction with highly expressed cancer target proteins
		receptor binding	molecular docking studies	
**8a-g**	A2780	cellular uptake	ICP-MS	noticeable cellular uptake with different Zn distribution profiles, induction of caspase-dependent apoptosis and mitochondria alterations, ROS overproduction, high ability to bind DNA through intercalation/groove binding,
		apoptosis	caspase-Glo® 3/7 assay	
		DNA binding	UV-vis titration experiments, fluorescence studies with EB and CD spectrophotometry	
		ROS generation	nitro blue tetrazolium (NBT) assay	

Compound	Cell lines	Mechanism analysis	Method	Main outcomes
		morphological analysis	transmission electron microscopy (TEM)	albumin binding that decreases the complex activity.
		albumin binding	fluorescence studies	
**12**	MDA-MB-231	apoptosis	Apo-TRACE assay	induction of cancer cell apoptosis by initiating the caspase cascade, delay in cellular migration, down-regulation of EMT-related genes
		cell migration	scratch assay	
		regulation of EMT markers	immunofluorescence studies	
**14a-j**		DNA binding	UV-vis titration experiments, fluorescence studies with EB and CD spectrophotometry, molecular docking studies	induction of cancer cell apoptosis, cell cycle arrest at G0/G1 phase, DNA interaction via intercalation into the base pairs of DNA
**15a-h**		DNA binding	fluorescence quenching and CD spectrophotometry	strong DNA interaction, critical role of halogen anions in the antitumor activity of complexes, interaction with highly expressed cancer target proteins
		protein binding	molecular docking studies	
**19a**	MCF-7	apoptosis	flow cytometry, nuclear staining with Hoechst 33342	induction of cancer cell apoptosis, Inhibition of cell cycle progression through G1 and G2/M phases.
		cell cycle analysis	flow cytometry	
**20**	HeLa	apoptosis	flow cytometry	DNA interaction via an intercalating mode, induction of cancer cell apoptosis, cleavage activity
		DNA binding	UV-vis titration experiments, fluorescence studies with EB, viscosity measurements, molecular docking studies	
		DNA cleavage	agarose-gel electrophoresis experiments	
**21a-b**	HeLa	apoptosis	flow cytometry, confocal microscopy	DNA interaction by insertion into a DNA base pair in relative parallel, induction of cancer cell apoptosis, cleavage activity
		DNA binding	UV-vis titration experiments, fluorescence studies with EB, molecular docking studies	
		DNA cleavage	agarose-gel electrophoresis experiments	
**22a-b**	SMMC-7721	apoptosis	flow cytometry, confocal microscopy	induction of cancer cell apoptosis by increase of intracellular ROS levels
		ROS generation	DCFH-DA fluorescence analysis	
**27**		protein binding	molecular docking studies	good affinity for PI3Kg
**28a**	SHSY5Y	apoptosis	flow cytometry, cell staining with Hematoxylin and Hoechst 33342	DNA interaction via a binding mode different from the classical intercalating mode
		cell cycle analysis	flow cytometry	

Compound	Cell lines	Mechanism analysis	Method	Main outcomes
		DNA binding	UV-vis titration experiments, fluorescence studies with EB	
**30a-b**	MDA-MB-231	apoptosis	flow cytometry	induction of cancer cell apoptosis
**32**		DNA binding	fluorescence studies with ethidium bromide (EB), CD spectrophotometry, viscometric measurements, molecular docking studies	DNA interaction via electrostatic binding mode
**33a**	P4, MES-SA, MES-SA/Dx5	cellular uptake	ICP-MS	noticeable cellular uptake, DNA interaction via intercalating and electrostatic binding mode
		DNA binding	fluorescence studies with ethidium bromide (EB), CD spectrophotometry	
**34**		DNA binding	UV-vis titration experiments, fluorescence studies with EB, molecular docking studies	DNA interaction, moderate DNA cleavage possibly via hydrolytic pathway
		DNA cleavage	agarose-gel electrophoresis experiments	
**38**	L5178Y	reversal of multidrug resistance (MDR)	flow cytometry	Inhibitory effect of the ABC-transporter PGP drug efflux pump
**39**	PC3, PNT1A	cellular uptake	fluorescence studies	noticeable cellular uptake, up-regulation of genes responsible for positive modulation of programmed cell death
		cancer genes expression	western blot analysis, quantitative RT-PCR	
**41**		DNA binding	UV-vis titration experiments, fluorescence studies with EB	DNA interaction via an intercalating mode, low SOD activity
**43**		DNA binding	UV-vis titration experiments	DNA interaction via an intercalating mode, DNA-cleavage activity, super oxide anion scavenging activity.
		DNA cleavage	agarose-gel electrophoresis experiments	
		superoxide dismutase (SOD) activity	NBT assay	
		protein binding	molecular docking studies	
